# A Novel Technique for Accelerated Culture of Murine Mesenchymal Stem Cells that Allows for Sustained Multipotency

**DOI:** 10.1038/s41598-017-13477-y

**Published:** 2017-10-17

**Authors:** Courtney M. Caroti, Hyunhee Ahn, Hector F. Salazar, Giji Joseph, Sitara B. Sankar, Nick J. Willett, Levi B. Wood, W. Robert Taylor, Alicia N. Lyle

**Affiliations:** 1 The Division of Cardiology, Department of Medicine, Emory University, Atlanta, GA USA; 2 The Department of Orthopaedics, Emory University, Atlanta, GA USA; 3 The Atlanta Veterans Affairs Medical Center Atlanta, Decatur, GA USA; 4 The Wallace H. Coulter Department of Biomedical Engineering, Georgia Institute of Technology and Emory University, Atlanta, GA USA; 5 George W. Woodruff School of Mechanical Engineering, Georgia Institute of Technology, Atlanta, GA USA; 6 Parker H. Petit Institute for Bioengineering & Bioscience, Georgia Institute of Technology, Atlanta, GA USA

## Abstract

Bone marrow derived mesenchymal stem cells (MSCs) are regularly utilized for translational therapeutic strategies including cell therapy, tissue engineering, and regenerative medicine and are frequently used in preclinical mouse models for both mechanistic studies and screening of new cell based therapies. Current methods to culture murine MSCs (mMSCs) select for rapidly dividing colonies and require long-term expansion. These methods thus require months of culture to generate sufficient cell numbers for feasibility studies in a lab setting and the cell populations often have reduced proliferation and differentiation potential, or have become immortalized cells. Here we describe a simple and reproducible method to generate mMSCs by utilizing hypoxia and basic fibroblast growth factor supplementation. Cells produced using these conditions were generated 2.8 times faster than under traditional methods and the mMSCs showed decreased senescence and maintained their multipotency and differentiation potential until passage 11 and beyond. Our method for mMSC isolation and expansion will significantly improve the utility of this critical cell source in pre-clinical studies for the investigation of MSC mechanisms, therapies, and cell manufacturing strategies.

## Introduction

Bone marrow-derived mesenchymal stem cells (MSCs) are a highly promising source for cell and gene therapy strategies. MSCs continue to generate interest for their use in therapeutic and translational applications because they can be used to directly generate multiple cell and tissue types and they also provide therapeutic benefits via paracrine signaling^1^. MSCs have been employed for therapeutic applications in settings ranging from musculoskeletal injury to cardiovascular disease and cancer. Pre-clinical MSC studies have demonstrated therapeutic promise and the number of clinical studies continues to increase; however, successful translation of MSCs as a widely available therapy remains a challenge. Major limitations preventing successful translation are the scalability of cell manufacturing protocols and differences in MSC potency across species and between individuals.

Mouse models remain one of the most powerful tools for performing mechanistic studies and preclinical testing of new therapeutics, including MSC based therapies. There are several published protocols for the isolation and culture of murine MSCs (mMSCs). These utilize a range of techniques from plastic adherence and Percoll gradients to immunodepletion; however, investigators continue to articulate difficulties in the isolation and expansion of mMSCs^2–5^. Common issues with mMSC culture include the inability to efficiently and reproducibly grow cells that maintain their multipotency and differentiation potential. Additionally, many protocols for mMSC generation select for rapidly dividing subpopulations, which may not produce representative or reproducible populations of MSCs for scientific investigation. Furthermore, the duration of time that it takes to generate a pure mMSC population in sufficiently large numbers to perform adequately powered studies is both lengthy (months) and laborious when using the most commonly accepted culture techniques^6–8^. Therefore, we set out to establish a highly reproducible way to isolate and culture murine MSCs that is both simple and effective and avoids common pitfalls commonly associated with the culture of MSCs.

MSCs can be isolated from various tissues including bone marrow, adipose tissue, and peripheral and cord blood, to name a few^9^. The therapeutic potential of MSCs is an active area of investigation and multiple cell types can be generated from MSCs including: osteoblasts, chondrocytes, tenocytes, adipocytes, and smooth muscle cells^1^. The method described herein utilizes the bone marrow as a source of MSCs. While MSCs have been successfully cultured from a number of mammalian species, we sought to develop a simple, straightforward technique for the isolation and culture of mMSCs that requires minimal MSC manipulation (sorting, enrichment, depletion, etc.) and minimizes the time from the bone marrow niche to plating, all while enhancing cell proliferation and differentiation potential. Previous studies have investigated a variety of culture conditions and supplementation techniques to enhance MSC proliferation. One promising approach for efficient MSC culture is to emulate the physiologic environment *in vivo* by growing MSCs under low oxygen tension (5% oxygen) conditions. Indeed, S. Boregowda, *et al*. and others have demonstrated that hypoxic conditioning significantly enhances MSC cell proliferation kinetics and alters metabolism^10–13^. A second approach to optimize stem cell proliferation in the culture setting is to supplement the culture media with growth factors. Basic fibroblast growth factor (bFGF) supplementation was previously linked to the maintenance of stemness and the suppression of senescence with an optimal effect at 10 ng/mL, making bFGF at this dose a viable growth factor candidate^14^. It was established recently by Fábián, *et. al*. that the combination of hypoxia and bFGF can improve human MSC proliferation and that this is mediated, in part, by ERK pathway activation and increased expression of HIF-1α^15^. Therefore, the use of hypoxia with bFGF supplementation could provide a promising approach to consistently generate mMSCs for pre-clinical mechanistic studies.

Herein, we describe a method to generate mMSCs that results in rapid expansion without cell senescence or a change in the multipotency with passage, which is in contrast to standard culture approaches currently employed. We found that low oxygen tension (5% O_2_ = hypoxia) in combination with bFGF supplementation (10 ng/mL) has an additive effect on mMSC proliferation, resulting in fast expansion, while simultaneously preserving stemness and suppressing senescence. This new approach generates mMSCs faster than existing protocols, is highly reproducible, allows MSCs to maintain their multipotency and differentiation potential beyond passage 11, and produces cells that demonstrate therapeutic potential *in vivo*.

## Results

### MSC Culture System Development and Characterization

The ability to reproducibly and efficiently expand mMSCs that maintain their multipotency and differentiation potential without transformation remains a difficult task. With a growing interest in the use of MSCs for various translational applications, we sought to develop a highly reproducible method to isolate and culture mMSCs that will allow investigators to generate MSCs from available transgenic mice for effectively studying the role of specific gene(s) or protein(s) of interest. We tested two biologically relevant conditions for their effects on mMSC proliferation independently and in combination: (1) the use of low oxygen tension, i.e. hypoxia, which emulates the bone marrow environment and (2) the use of bFGF as a stimulus to preserve stemness and deter senescence.

**Figure 1:**
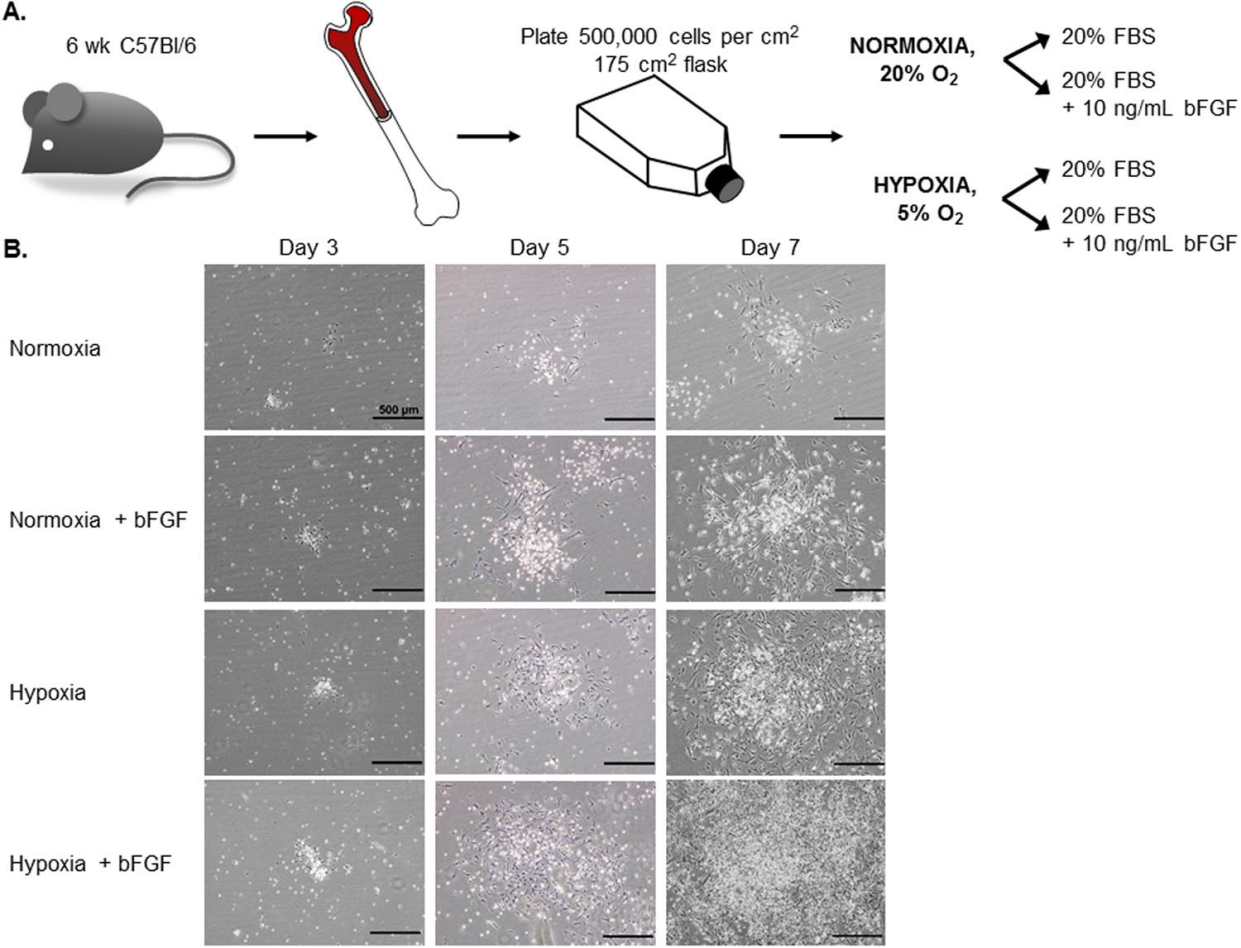
MSC Culture Condition Development. (**A**) Schematic illustrating the workflow and conditions used to optimize the culture of mMSC. The fresh isolated bone marrow cells were divided into 4 groups: normoxic (20% O_2_) or hypoxic (5% O_2_) conditions +/−10 ng/mL of bFGF supplement. (**B**) The representative phase contrast images were acquired (10x) at different time points during the p0 to p1 growth phase: d3, d5, and d7 post-plating. (d14 images found in Fig. 2A). Scale bars = 500 μm.

We utilized 6 week old C57Bl/6 mice for optimization of this method. The bone marrow from the diaphysis of each hindlimb long bone was flushed and viable cells were rapidly plated at a density of 500,000 cells/cm^2^. After initial plating, cells were subdivided into the following groups to test the effects of oxygen tension, bFGF supplementation, and the combination of these two conditions on MSC cell growth: (1) Normoxia, (2) Normoxia + 10 ng/mL bFGF, (3) Hypoxia, and (4) Hypoxia + 10 ng/mL bFGF (Fig. 1A).

**Figure 2:**
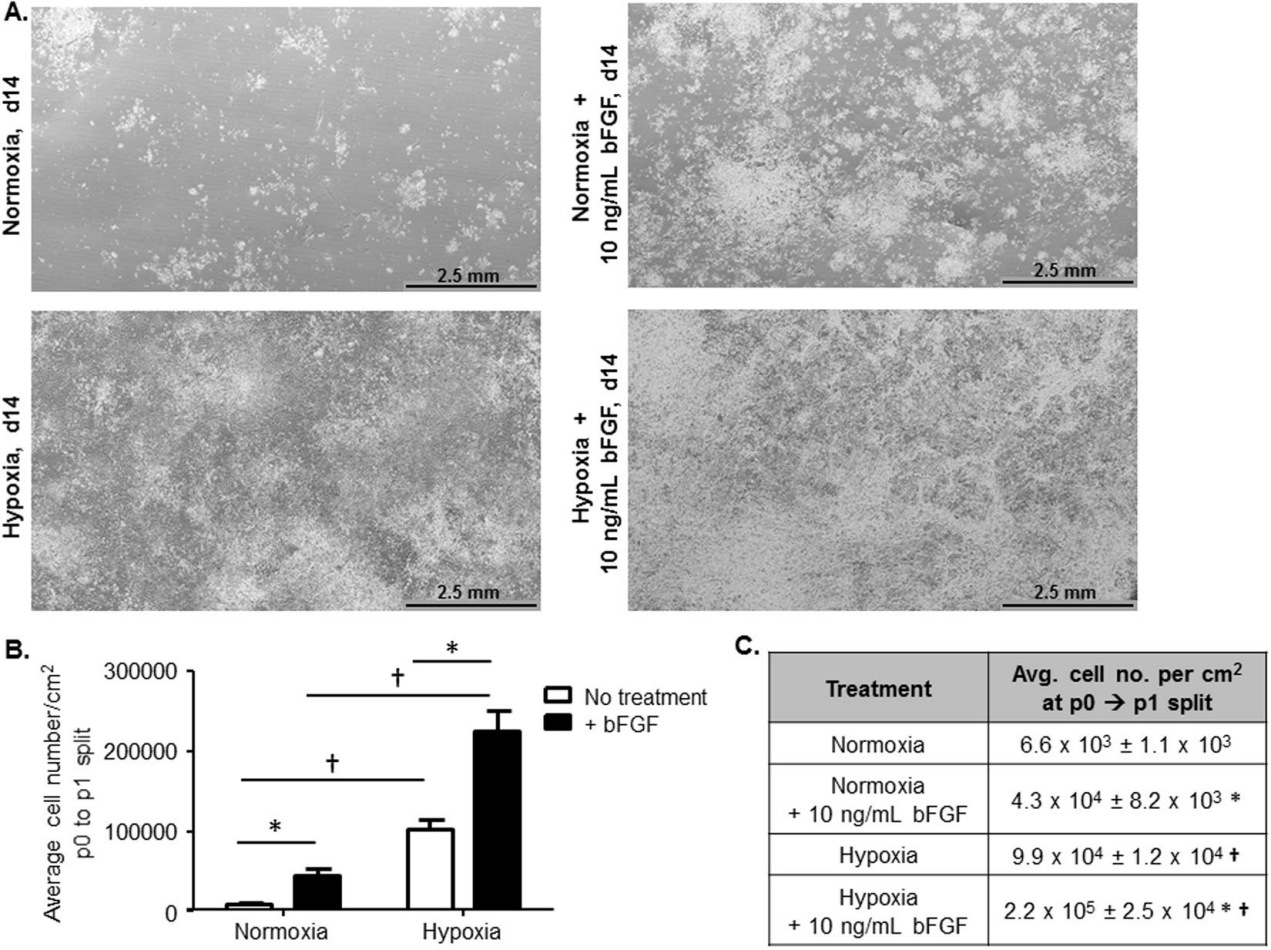
Increasing Cell Number per cm^2^ Under Different MSC Culture Conditions. (**A**) 30 continuous phase contrast images were acquired and stitched together to demonstrate MSC colony size and density in each of the culture conditions. Scale bar = 2.5 mm. (**B**) Average cell number/cm^2^ was calculated when each of the culture conditions reached similar points of surface area coverage that were appropriate for passaging (n = 5–8 independent cell lines; *p < 0.001 vs. no treatment (white bars) and ^†^p < 0.0001 vs. normoxia). (**C**) Table reports the average cell number/cm^2^ with S.E.M. for n = 5–8 independent cell lines; *p < 0.001 vs. no treatment and ^†^p < 0.0001 vs. normoxia; data presented as mean ± S.E.M.

MSC colony growth was monitored over time and phase contrast images were acquired at 3, 5, 7, and 14 days post-plating to document colony size and density differences across the experimental groups. While no differences were observed at day 3, colony sizes were different as early as day 5, by qualitative assessment, and these differences were further amplified with time (days 7 and 14; Figs 1B and 2A, and Supplemental Fig. 1B). The reduction of oxygen tension from 20% to 5% resulted in increased mMSC colony number, size and cell density. 10 ng/mL bFGF supplementation also resulted in increased colony number, size and cell density. Notably, when hypoxia and bFGF treatments were used in combination, we observed a striking increase in apparent colony size and the colonies appeared denser, suggesting that hypoxia and bFGF supplementation promote a synergistic effect on MSC proliferation (Figs 1B and 2A).

To further validate and quantify these differences, when cells reached a similar level of surface area coverage we quantified the number of viable cells per cm^2^ in each condition at the time of p0 to p1 split for n = 5–8 independently generated murine MSC cell culture lines. The average cell number per cm^2^ was significantly increased by bFGF supplementation or hypoxia alone (Fig. 2B and 2C); however, the combinatorial use of hypoxia and bFGF generated an extraordinary synergistic increase in cell number per cm^2^ that equated to a ~33-fold increase compared to standard normoxic conditions alone.

### Enhanced MSC Proliferation with Reduced MSC Senescence

**Figure 3:**
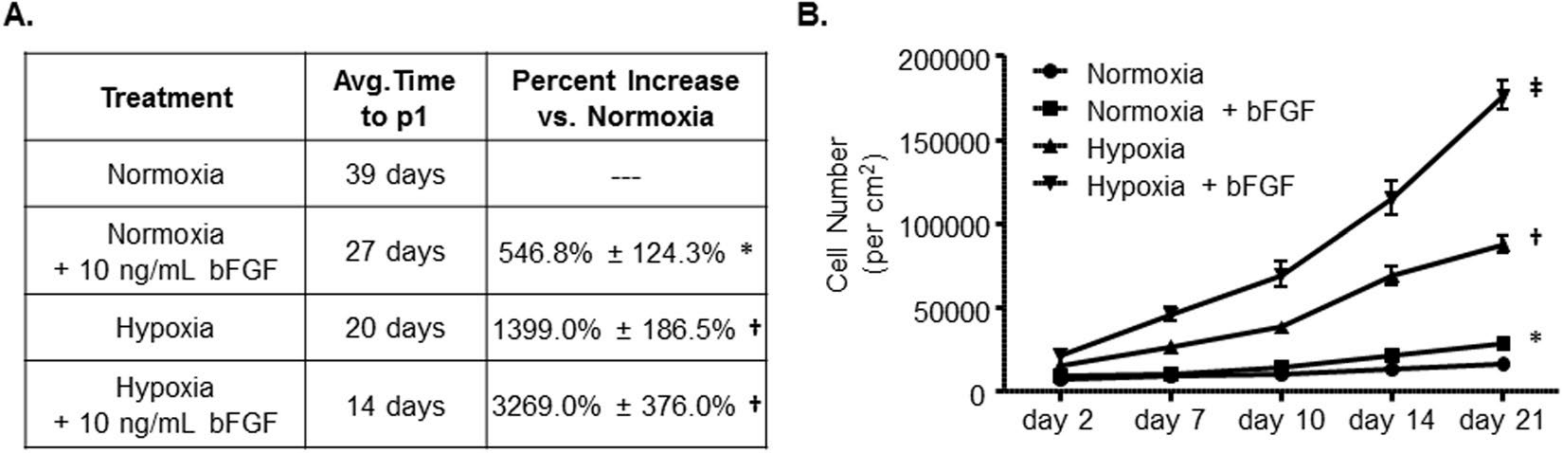
Increased MSC Proliferation Rates and Decreased Time to Passage 1. (**A**) The average number of days to reach similar levels of surface area coverage and for passaging from p0 to p1 and the average percent (%) increase in cell number/cm^2^ across the different culture conditions compared to classic normoxia conditions. n = 5–8; *p = 0.002 vs. normoxia, ^†^p < 0.0001 vs. normoxia. (**B**) Proliferation assay for each culture condition. n = 5–8; *p < 0.001 normoxia vs. normoxia+bFGF at d14 and d21; ^†^p < 0.001 vs. normoxia and normoxia+bFGF; ^‡^p < 0.001 vs. normoxia and normoxia+bFGF, ^‡^p < 0.005 vs. hypoxia; data presented as mean ± S.E.M.

MSCs cultured in normoxia alone took 39 days on average to reach a level of surface area coverage necessary for passage. In Fig. 2A, the use of bFGF supplementation and hypoxia alone increased the observed cell density and colony frequency in comparison to normoxia. To quantify the effects of hypoxia and bFGF supplementation on MSC cell numbers, we assessed how hypoxia and bFGF affected cell numbers at the time of p0 to p1 split (Fig. 3A). Normoxic cells supplemented with bFGF exhibited a 546.8 ± 124.3% increase in cell number compared to normoxia, reducing the time to reach optimal surface area coverage to 27 days. Hypoxia alone increased cell numbers by 1,399 ± 186.5% compared to normoxia and reduced the average time to optimal surface area coverage to 20 days. The combination of hypoxia and bFGF supplementation increased the average cell number by 3,269.0 ± 376.0% and decreased the time for cells to reach optimal surface area coverage to 14 days. Overall, the average time to reach p3, when MSC populations are commonly characterized for *in vitro* and *in vivo* applications, was reduced from 2-3 months to 1 month with hypoxia+bFGF conditions (Fig. 3A)

A standard proliferation assay was utilized and viable cell numbers were quantified at 2, 7, 10, 14, and 21 days post-plating. bFGF supplementation of normoxic MSCs increased cell proliferation compared to normoxia alone at days 14 and 21 (Fig. 3B). Hypoxia significantly increased cell proliferation as early as day 7 compared to normoxia and the effect of hypoxia increased over time compared to normoxia alone and normoxia+bFGF. The effects of hypoxia and bFGF supplementation were found to dramatically and synergistically increase cell proliferation compared to all other treatment groups at day 7 and these differences increase over time. These data were consistent with Figs 1B and 2. Similar proliferation rates are still observed in hypoxia and hypoxia+bFGF cultured MSCs as far out as passage 12 (Supplemental Fig. 2A), at which point normoxia and normoxia+bFGF MSCs have senesced.

**Figure 4:**
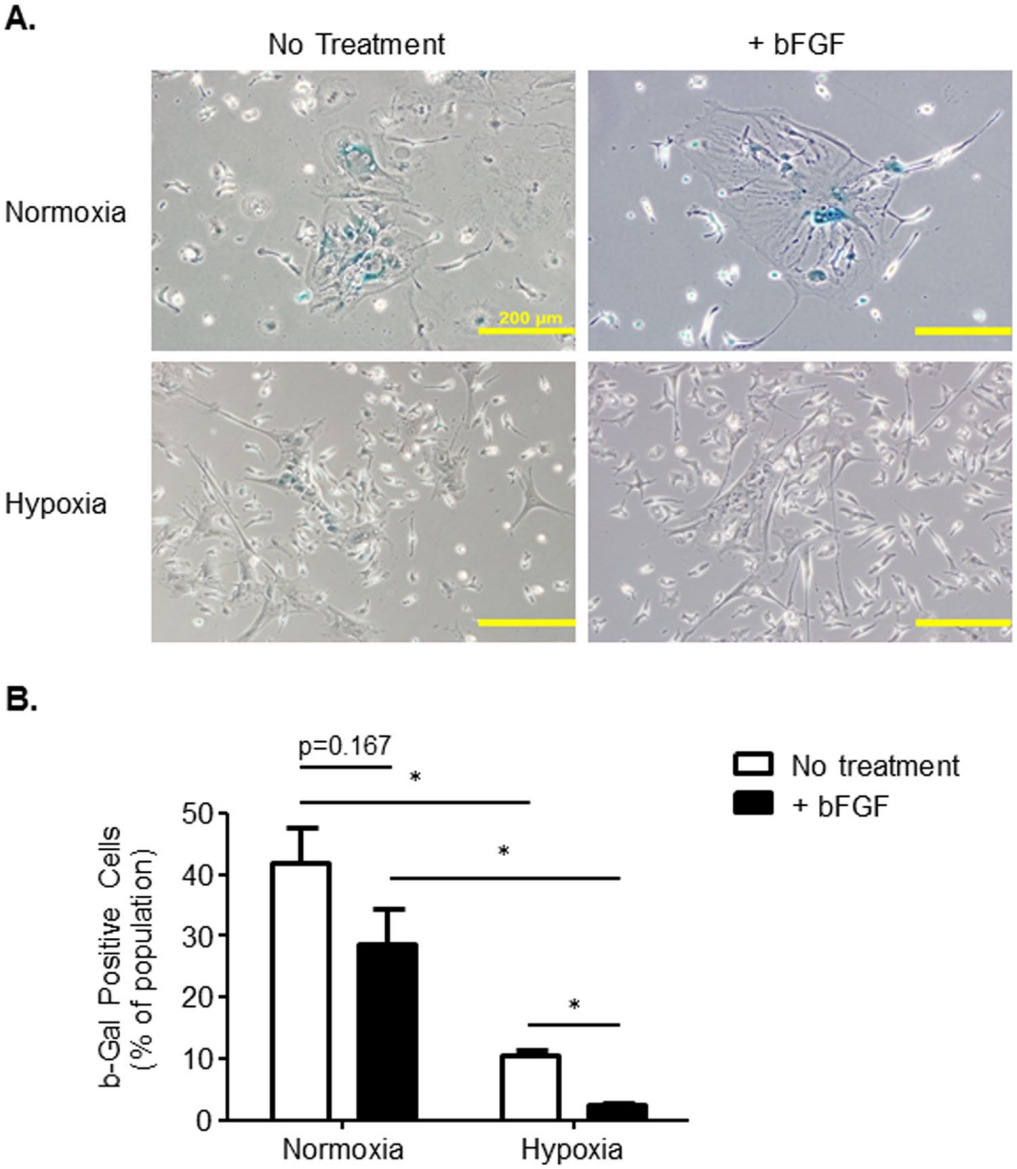
Oxygen Tension and bFGF Supplementation Differentially Effect Senescence. (**A**) Representative phase contrast images (10x) of β-Gal staining for 4 different groups of cells. Scale bars = 200 μm. (**B**) % of β-Gal positive cells within a culture population. Image J software was used to quantify and analyze the stitched images and count the number of β-Gal positive cells and the total number of cells. n = 3; *p < 0.01; data presented as mean ± S.E.M.

Challenges with standard mMSC culture protocols include senesce at early passages and transformation into immortalized cells. Senescence-associated β-galactosidase (SA-β-Gal) activity is a well-known biomarker for senescence that can be detected with x-gal treatment and blue staining indicates senescence^16^. To determine if oxygen tension or bFGF supplementation influence cell senescence, mMSCs were incubated with x-gal and blue-stained cells were quantified. Hypoxia and hypoxia+bFGF MSC cultures had fewer large, senescent, β-Gal positive cells compared to normoxia or normoxia+bFGF MSC cultures (Fig. 4A). Additionally, the smaller MSCs in close proximity to these large senescent cells also stained positive for senescence. To quantify the β-Gal positive cells in each culture condition, we counted the number of blue cells and calculated the % of β-Gal positive cells present in each condition. Hypoxia significantly decreased β-Gal positive staining from 46.0% in normoxia to 10.6% (Fig. 4B). bFGF supplementation decreased cell senescence further in hypoxic MSCs compared to hypoxia alone; however, this bFGF effect did not reach statistical significance, despite trending, under normoxia conditions. The culture group that demonstrated the least senescence was the hypoxia+bFGF MSC group, where the mean percentage of β-Gal positive cells was significantly reduced to ~5%. Thus, hypoxia conditioning decreases cell senescence and bFGF supplementation further reduces senescence under hypoxic conditions.

We suggest removing any residual large senescent cells and the underlying small senescent cells that remain in the culture by (Fig. 4A) using the trypsin depletion method outlined in Supplemental Fig. 1. In doing so, we observed the cell size variability that can persist in murine MSC cultures was minimized in 3 out of 4 cell culture groups. Removal of the senescent cells decreased the number of cells that exhibit increased granularity (Q1 + Q2), increased size (Q2 + Q3), or both (Q2 only) in MSC cultures (Supplemental Fig. 3A and 3B). Furthermore, the depletion of senescent cells resulted in MSC cultures where the majority of the cells exhibited a consistent size and ~86.8% fell within the gated population (Q4; Supplemental Fig. 3A).

### MSC Immunophenotype and Multipotency

**Figure 5:**
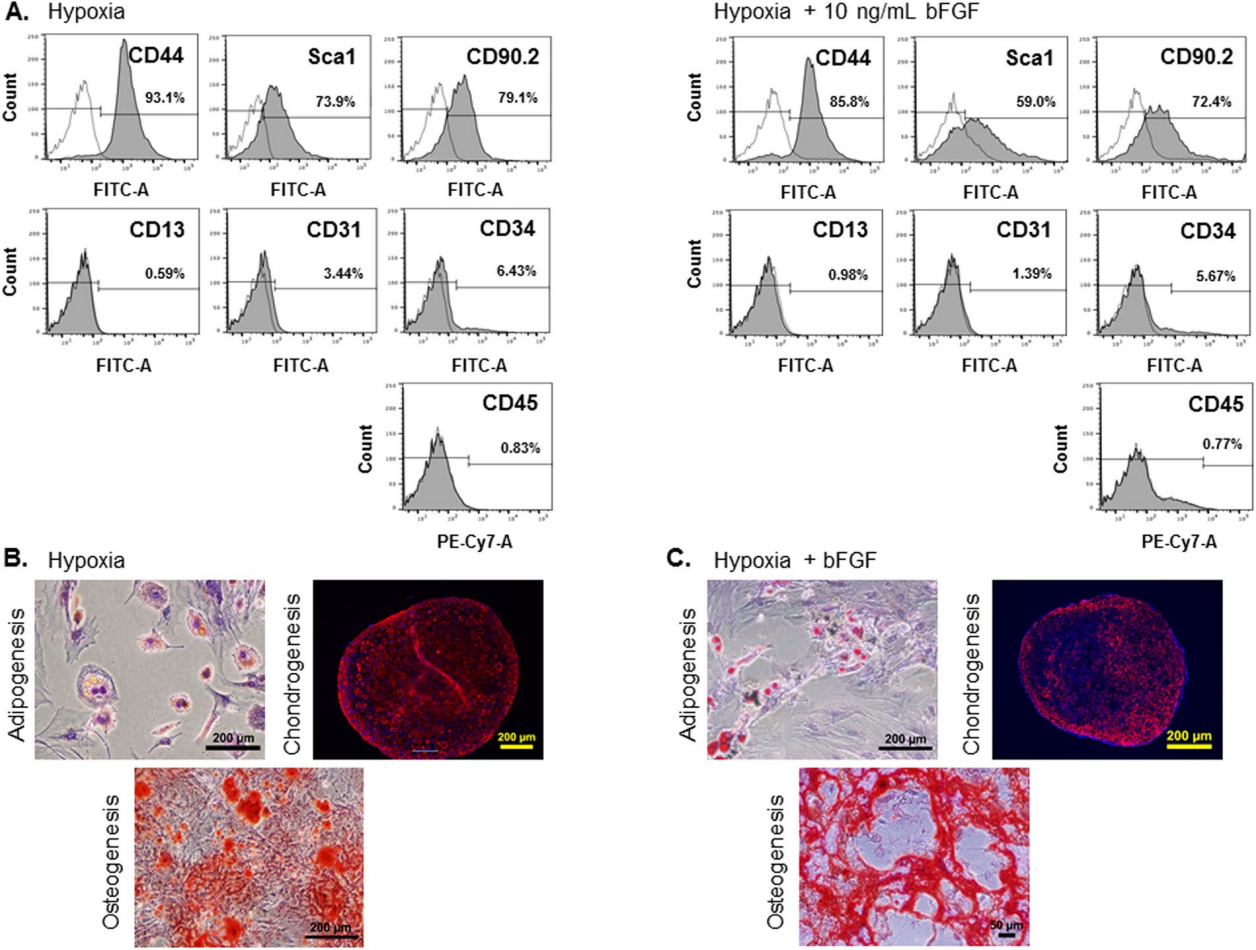
Immunophenotyping MSCs by FACS Analysis and Verification of Multipotency by Differentiation. (**A**) p3–4 of mMSCs detected positive for the MSC markers CD44, Sca1 and CD90.2, and negative for the CD34, CD45, CD13, and CD31. (**B** and **C**) To validate multipotency, hypoxia and hypoxia+bFGF MSCs were induced to differentiate into adipocytes, chondrocytes, or osteoblasts. After differentiation, cells were stained as follows to confirm successful differentiation: (1) adipocytes were stained with Oil Red O, scale bars = 200 μm; (2) chondrocytes were stained with an antibody for Collagen II, scale bars = 200 μm and (3) osteoblasts were stained with Alizarin Red for calcium, scale bar = 200 μm (5B) and 50 μm (5C). Representative images for each cell type and differentiation condition are shown.

MSC phenotype and multi-lineage differentiation potential were investigated to determine stem cell specific properties. To verify that our murine MSC cultures were pure MSC populations devoid of other cell types commonly found in the marrow, we utilized flow cytometry analysis. Immunophenotyping profiles for mMSCs grown in normoxic conditions have been described and our mMSCs grown in normoxia and normoxia+bFGF immunophenotyped as previously reported (data not shown)^6,7,17^. To validate that mMSCs cultured in hypoxia and hypoxia+bFGF have a similar cell surface marker profile to cells cultured in normoxia, mMSCs from these 2 conditions were stained at p3-p4 with each of the antibodies outlined in Table 1. Flow cytometry analysis showed that mMSCs from hypoxia and hypoxia+bFGF exhibit the same cell surface marker profile as MSCs cultured under normoxic conditions (Fig. 5A). Hypoxia and hypoxia+bFGF mMSCs stained positive for the mesenchymal lineage expression markers CD44, Sca-1, and CD90.2, while staining negative for markers of hematopoietic cells (CD45), monocytes (CD13), mature endothelial cells (CD31), and circulating endothelial progenitor and hematopoietic progenitor cells (CD34).

**Table 1:** Surface Marker Antibodies used for FACS Immunophenotyping.

Cell Type	Target	Antibody Clone	BD Catalog #	Fluorochrome	Isotype
MSC+	CD44	IM7	561859	FITC	Rat IgG2b,k
MSC+	Sca-1	E13–161.7	553335	FITC	Rat IgG2a,k
MSC+	CD90.2	30-H12	553013	FITC	Rat IgG2b,k
Hematopoeitic	CD45	30-F11	552848	PE-Cy7	Rat IgG2b,k
Monocytic	CD13	R3-242	558744	FITC	Rat IgG1, k
Endothelial	CD31	390	558738	FITC	Rat IgG2a,k
EPC + (MSC+/−)	CD34	RAM34	553733	FITC	Rat IgG2a,k
Isotype Control	Rat IgG2a,k	R35-95	553929	FITC	
Isotype Control	Rat IgG2b,k	A95-1	553988	FITC	
Isotype Control	Rat IgG2b,k	A95-1	552849	PE-Cy7	
Isotype Control	Rat IgG1, k	R3-34	553924	FITC	

Murine MSCs were immunophenotyped using fluorescence-activated cell sorting (FACS) analysis for the presence of the MSC markers CD44, Sca-1, and CD90.2 and the absence of the endothelial, monocytic, and hematopoietic markers CD31, CD13, and CD45, respectively. The BD Bioscience antibodies used for MSC immunophenotyping and the isotype controls used for these FACS experiments, as well as antibody clone number, catalog number, and fluorochrome are included in the table shown. FITC = fluorescein isothiocyanate; PE = phycoerythrin.

To assess multipotency, hypoxia and hypoxia+bFGF mMSCs were differentiated into three different mesenchymal lineages: adipocytes, chondrocytes and osteoblasts (Fig. 5B and 5C). In both groups, the small lipid vacuoles indicative of adipogenesis appeared at day 3 after induction and continuously matured over time. These intracellular lipid droplets stained positive with Oil red O reagent, an indicator of adipogenesis. Separately cells were cultured under osteogenic conditions and extracellular calcium accumulation, a widely utilized indicator for osteogenesis, was confirmed by Alizarin Red staining. mMSCs under both conditions showed an abundant deposition of calcium. For the chondrogenic differentiation assay, 2–3 weeks after chondrogenic induction, chondrogenic pellets were stained with a type II collagen antibody and showed expression throughout the pellet. These results demonstrate that mMSCs cultured under hypoxic conditions classify as mesenchymal stem cells by cell surface marker expression and properly undergo tri-lineage differentiation. Furthermore, bFGF supplementation does not affect these mesenchymal stem cell properties.

### Multivariate Analysis of Extra- and Intra-Cellular Signaling

**Figure 6:**
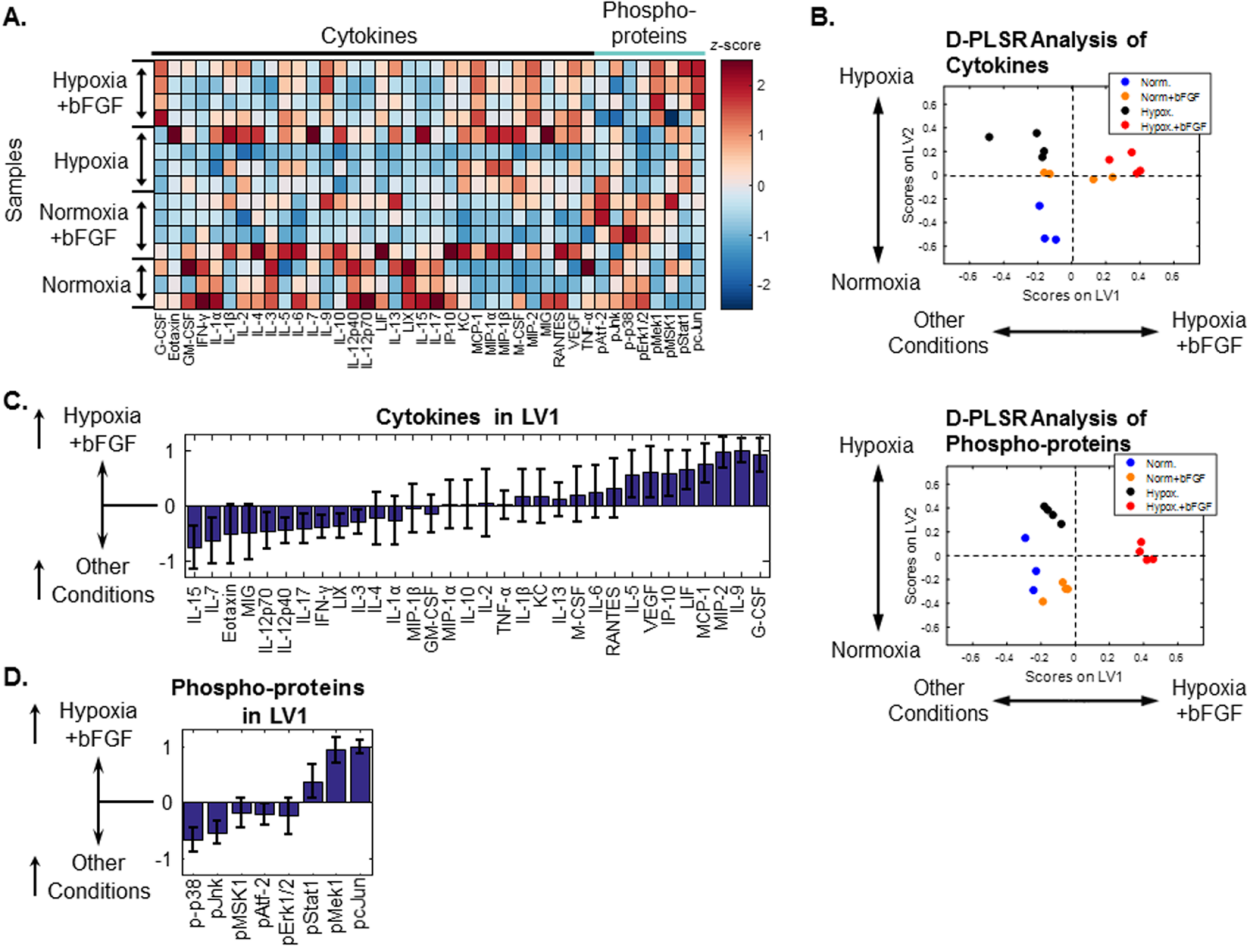
Culture conditions modulate MSC extra- and intra-cellular signaling. (**A**) Quantification of 32 cytokines in culture media and 8 phospho-proteins in cell lysates from MSCs cultured under four growth conditions for 7 days (multiplexed immunoassay, Millipore). All data were normalized to total protein in cell lysates and z-scored along each column. n = 3–4 independent cultures. (**B**) Multivariate discriminant partial least squares regression (D-PLSR) analysis segregated growth conditions based on either cytokine expression or phosphorylation of intracellular signaling proteins. Each analysis separated hypoxia+bFGF conditions to the right, normoxia toward the lower left and hypoxia toward the upper left. (**C**) The cytokine analysis separated samples along LV1 in terms of a linear combination of cytokines that correlate (positive values) or inversely correlate (negative values) with the hypoxia+bFGF condition. Error bars on each cytokine were generated by Monte Carlo sub-sampling the data set 1000 times, randomly removing 20% of the samples in each iteration and re-generating the LV1 profile each time (mean ± SD). (**D**) The phospho-protein analysis separates samples along LV1 in terms of a linear combination of phospho-proteins (mean ± SD, 1000 Monte Carlo iterations, 20% sample removal).

MSC proliferation and senescence are regulated, in part, by autocrine and intracellular phospho-protein signaling. To characterize how MSC cytokine production and signaling shifts in response to hypoxia or bFGF treatment, we utilized multiplexed immunoassays to quantify expression of 32 cytokines secreted by MSCs into the culture medium and phosphorylation of 8 intracellular signaling proteins within the mitogen activated protein kinase (MAPK) pathway from cell lysates (Fig. 6A). To account for the different proliferation rates under each culture condition, cytokine measurements were normalized to total protein, as quantified via Pierce BCA and phospho-protein measurements were normalized by loading an equal amount of total protein into each assay well and normalized cell lysate loaded into the multiplexed assay to total protein.

To account for the multidimensional nature of the data, we used a discriminant partial least squares regression (D-PLSR)^18^ to identify *profiles* of cytokines and phospho-proteins that segregated hypoxia+bFGF-conditioned MSCs from our three other culture conditions. Both cytokines and phospho-proteins were able to cluster hypoxia+bFGF treated wells to the right, while other conditions clustered toward the left (Fig. 6B). Our analysis revealed that multiple cytokines were strongly correlated with the hypoxia+bFGF treatment condition (Fig. 6C), including G-CSF and IL-9 as the top two correlates. These cytokines are reported to promote proliferation^19^ and survival^20^ of multiple cell types and thus represent possible autocrine signaling mechanisms by which hypoxia+bFGF promotes MSC proliferation. In contrast, application of hypoxia alone increased expression of MCP-1 and MIP, which have chemotactic properties (Supplemental Fig. 5A). The phospho-protein D-PLSR (Fig. 6B) identified increased cJun phosphorylation as the top correlate in the hypoxia+bFGF condition (Fig. 6D). Activation of cJun by phosphorylation promotes cell survival and proliferation and inhibits senescence^21^. In contrast, hypoxia alone only yielded an increase in phosphorylation of Stat1 (Supplemental Fig. 5B, which is primarily known for its inflammatory properties^22^. Phospho-Mek1 is the second strongest correlate with hypoxia+bFGF and is also known to positively affect proliferation^23^. Our data also demonstrate reduced steady-state phosphorylation of p38, which has been implicated in human endometrium-derived MSC senescence^24^. Furthermore, phospho-Jnk is also down-regulated in hypoxia+bFGF treated MSCs, which is un-expected, since it is the primary kinase for cJun, and is thus canonically coordinately regulated. Together, these extra- and intra-cellular signaling data suggest that hypoxia and bFGF co-operate to provide a complex shift in extra- and intra-cellular signaling, which promotes proliferation and reduces senescence.

### Sustained Multipotency and *In Vivo* Efficacy

**Figure 7:**
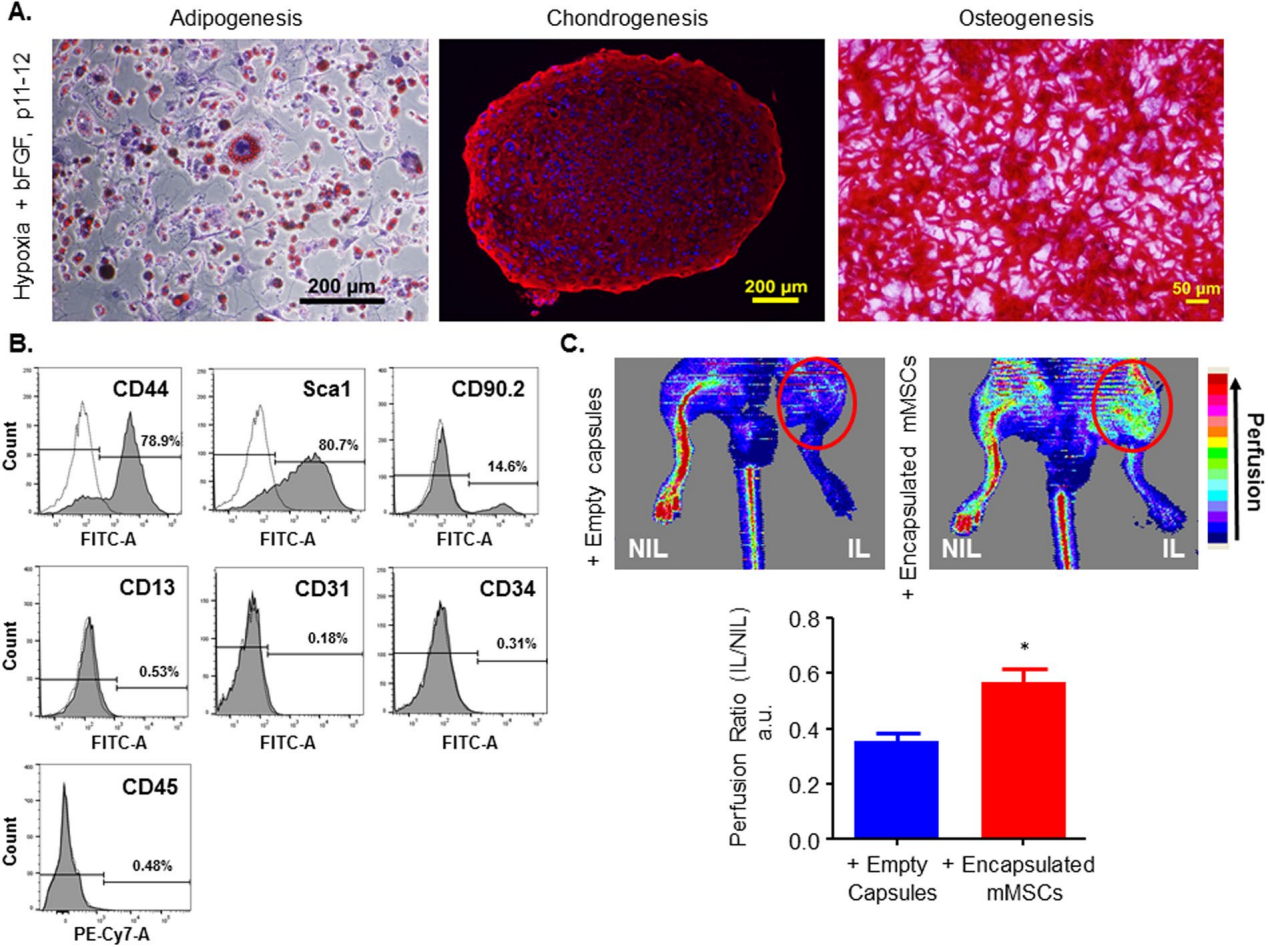
Sustained MSC Multipotentcy and *in vivo* Efficacy at Late Passages. (**A**) To verify that the MSCs retain multipotency at later passages (p11 and beyond), cells were stimulated to undergo tri-lineage differentiation and were evaluated. Adipogenesis and Chondrogenesis scale bars = 200 μm; Osteogenesis scale bar = 50 μm. (**B**) FACS histograms of p12 mMSCs grown in hypoxia+bFGF show that cells retain MSC specific surface marker expression and are positive for CD44, Sca1 and are negative for CD34, CD45, CD13, and CD31. At higher passages, however, cells have lost CD90.2 surface expression. (**C**) To test if late passage murine MSCs can promote and rescue neovascularization in a murine model of hindlimb ischemia, mMSCs (p11) were encapsulated in alginate and delivered to the ischemic hind limb (IL) by subcutaneous implantation. Laser Doppler perfusion imaging was performed at day 14 on animals that received either empty capsules or encapsulated mMSCs. Representative heat maps are presented to indicate perfusion levels. Perfusion levels were quantified for n = 3 animals per group and presented as a ratio of the perfusion level in the ischemic limb (IL) to the contralateral control non-ischemic limb (NIL). n = 3; *p = 0.02 vs. empty; data presented as mean ± S.E.M.

To determine if mMSCs cultured under hypoxia or hypoxia+bFGF conditions resist senescence and immortalization and maintain their differentiation potential until later passages, mMSCs were passaged further and cultured under the same conditions in which they were initially grown. MSC proliferation, phenotype, differentiation and apoptosis were all assessed again at passages 10-12. As shown in Fig. 7A, mMSCs grown under hypoxia+bFGF maintained differentiation potential at late passages (p11 shown). Furthermore, immunophenotyping results by FACS show that late passage MSCs (p12) grown under hypoxia+bFGF conditions are still positive and negative for appropriate MSC surface markers (Fig. 7B), with the exception of the loss of CD90.2 expression. Furthermore, upon serum withdrawal, these cells undergo apoptosis (not shown), suggesting that these cells are not immortalized. We also demonstrate that the enhanced proliferation rates and CFU promoted by culturing MSCs in hypoxia or hypoxia+bFGF conditions are sustained out to p9-12 (Supplemental Fig. 2A, B).

Landazuri N, *et. al*. previously demonstrated that MSCs grown in normoxic conditions were able to drive neovascularization in a murine model of hindlimb ischemia, as assessed by increased perfusion using Laser Doppler perfusion imaging (LDPI), when encapsulated and delivered subcutaneously^25^. This encapsulation approach also proved to be effective to deliver viable human MSCs in a rat model of myocardial infarction^26^. To determine if mMSCs grown in hypoxia+bFGF retained the therapeutic ability to improve neovascularization, and thus perfusion, in a murine model of hindlimb ischemia, we used a similar model. Encapsulated mMSC viability was confirmed using live/dead stain (Supplemental Fig. 4A, 4B) before *in vivo* delivery, as described previously^25,26^. Osteopontin knock-out (OPN−/−) animals normally exhibit impaired collateral vessel formation^27^; therefore, we used these animals in combination with a hindlimb ischemia model and evaluated if encapsulated mMSCs could rescue perfusion. Representative LDPI heat maps from OPN−/− animals treated with empty capsules or encapsulated mMSCs (p11) are shown (Fig. 7C). Encapsulated mMSCs grown under hypoxia+bFGF conditions were able to significantly rescue collateral vessel formation in OPN−/− animals, as assessed by an increase in perfusion measured by LDPI (day 14), compared to empty capsule treated animals.

Altogether, our results demonstrate that growing cells in 5% O_2_ (hypoxia) + 10 ng/ml bFGF efficiently generates mMSC cultures without a 3 month waiting period to reach p3 for immunophenotyping and differentiation validation and use for *in vivo* and *in vitro* applications. This new approach generates MSCs faster than existing protocols, is highly reproducible, and allows MSCs to maintain their multipotency and differentiation potential until passage 11, and still demonstrate therapeutic potential *in vivo* at these late passages.

## Discussion

Some of the most commonly utilized and highly cited methods to isolate and expand murine MSCs have proven effective^2,4–7,13^; however, the amount of time it takes to generate a pure MSC culture and obtain enough cells for *in vitro* and *in vivo* experiments is time consuming, expensive and results in decreased stemness and increased cell senescence. Furthermore, the ability to culture mMSCs in a simple, effective, and timely fashion from existing transgenic mouse models would fill a significant unmet need by allowing researchers to investigate the function of specific genes in various translational and differentiation processes in the pre-clinical lab setting. In this manuscript, we demonstrate that when MSCs are cultured from initial plating in 5% hypoxia and 10 ng/mL of bFGF together, they have dramatic synergistic effects. Our combinatorial method substantially increased the average number of mMSCs per cm^2^ (Fig. 2), improved the proliferation rate by >3,000% compared to normoxic conditions (Fig. 3), and allowed cells to reach p1 in 14 days, as opposed to the traditional method’s 4–6 weeks. Thus, we have minimized the time to reach p1 from p0 from an average of 5.5 weeks with normoxic conditions to 2 weeks in culture when grown in hypoxia+bFGF (Fig. 3). Not only did MSCs grow faster with hypoxia and bFGF combined, but the cultures were more representative MSC population compared to clonally expanded MSCs because all MSCs are utilized, not just a subset of rapidly dividing MSCs. Our data indicate that our hypoxia+bFGF culture strategy enhances proliferation potential while maintaining stemness as defined by ISCT standards^28^. However, MSCs take on a spectrum of phenotypes and gene expression profiles. Whether these different sub-populations possess different differentiation potentials remains an open research question. Delineation of the effects of different culture conditions on the distribution and differentiation potential of these sub-populations may be a fruitful avenue of future research. The culture method described in this manuscript should provide improved proliferative and multipotent consistency from one isolation to the next and between investigators and independent laboratories, thus increasing reproducibility in the field. MSCs grown using this method maintained their multipotency, as shown by their ability to undergo tri-linage differentiation (Fig. 5B, 5C), showed no differences in cell surface marker expression (Fig. 5A), and exhibited a marked reduction in senescence (Fig. 4) compared to previously reported methods for the culture of mMSCs^6,17^. Furthermore, we demonstrated that MSCs isolated and expanded with our combinatorial approach exhibit differences in their underlying cell signaling and cytokine production (Fig. 6) and their functional ability to promote *in vivo* neovascularization, even at a high passage (Fig. 7).

Murine MSCs are classically more difficult to expand in culture than human or rat MSCs^4^. Cultures of mMSCs are often contaminated by hematopoietic cells and, while immunodepletion can be used to remove hematopoietic precursors, the cultures often have additional issues^3^. Other mMSC protocols detail expansion from fast growing single colonies and are, therefore, not necessarily a representative population of MSCs. Our combinatorial technique utilizes the entire MSC cell population, not just rapidly dividing cells, to overcome some of the common pitfalls associated with murine MSC culture and expansion. The presence of senescent MSCs, if allowed to persist in culture, will limit the number of passages that the MSCs will grow. Therefore, we additionally present a depletion step whereby these senescent cells are removed from the MSC culture at the time of cell passage using our trypsin depletion method (Supplemental Fig. 1; see Methods for details). By removing these large senescent cells, and the underlying small senescent cells (Fig. 4A), we found that we were able to minimize cell size variability in 3 out of 4 culture groups by depleting cells with increased granularity (Q1 + Q2), increased size (Q2 + Q3), or both (Q2 only) in MSC cultures (Supplemental Fig. 3A and B). The depletion of senescent cells resulted in MSC cultures with a more consistent cell size (~86.8% within the expected gated population; Q4; Sup. 3A) and improved mMSC population purity. Additionally, the combinatorial approach of using hypoxia (5% O_2_) and bFGF decreased the time it takes to generate a large number of mMSCs (p0 to p1) from a traditional 5–6 week period (normoxia alone)^7^ to 14 days, increased cell number and density, and minimized the presence of senescent cells.

Oftentimes mMSCs immortalize and lose their multipotency and differentiation potential and thus behave more like cancer/tumor cells^29,30^. Additionally, mMSCs grown in normoxic conditions often senesce around p8–10^14,31^. We demonstrated that mMSCs cultured in hypoxia+bFGF are expandable to at least p12 with maintained multipotency, verified through their ability to undergo tri-lineage differentiation. Our data suggest that the mechanism(s) underlying the significant MSC expansion improvements in hypoxia+bFGF conditions is due to modulation of extra- and intra-cellular signaling that promote proliferation and reduce senescence (Fig. 6). To facilitate interpretation of the 32 cytokines and 8 phospho-proteins in our analysis, we used a multivariate statistical tool, a discriminant partial least square regression^18^, to identify profiles of extra- and intra-cellular signals that correlated with hypoxia+bFGF conditioning. While our analysis identified varying degrees of up- or down-regulation of each cytokine, we identified G-CSF, IL-9, and MIP-2 as the top positive cytokine correlates with hypoxia+bFGF conditioning. G-CSF and IL-9 are reported to promote proliferation and survival, respectively^19,20^. Additionally, MIP-2 (CXCL2) gene expression is reported to be down-regulated in senescent human MSCs^32^. Together, these data suggest that hypoxia+bFGF promotes proliferation and survival, and decreases senescence via a paracrine signaling mechanism.

Our intracellular signaling data identified phospho-cJun and phospho-Mek1 to be the top intracellular signaling correlates with hypoxia+bFGF at 7 days (Fig. 6C). Both of these phospho-proteins canonically promote cell survival, and cJun has been reported to reduce senecence^21,23^. In addition, phospho-p38 and phospho-Jnk were the top two negative correlates with hypoxia+bFGF. Down-regulated phosphorylation of p38 is consistent with prior work linking p38 signaling to endometrium-derived MSC senescence^24^. Since Jnk is the canonical kinase for cJun, it was unexpected to find them inversely varying in our data. Additionally, one recent study reported that hypoxia+bFGF increases Erk activation^33^, our findings are not in line with these. We hypothesize that the differences in Erk activation could potentially be a product of: 1) duration of hypoxia exposure at the time of Erk activation measurement (minutes to hours vs. days), 2) level of hypoxia (1% vs. 5%), 3) differences in bFGF dose (1 ng/mL vs. 10 ng/mL), or 4) species differences (human vs. mouse) in response to hypoxia+bFGF. We note, importantly, that the data presented here are taken at a single 7 day time point. Further studies are required to determine if pJnk or pErk were activated at earlier time points in the culture process, but are no longer activated at day 7. While murine MSCs are a valuable research tool, there is still a critical need for a scalable method to isolate and expand human MSCs for use in the clinic as cell based therapies. Fábián, Z. *et al*. have demonstrated that the use of hypoxia and bFGF together increases proliferation rate, but if growing human MSCs in hypoxia+bFGF conditions reduces senescence and extends passage numbers while retaining tri-lineage differentiation requires further investigation^15^.

The therapeutic potential of MSCs has been demonstrated in many environments^34–37^ and a body of research suggests that MSCs have therapeutic applications in cardiovascular disease pathologies^38–40^. Importantly, by expanding our understanding of MSC biology, we may be able to further refine MSC culture practices to enhance therapeutic applicability. Culturing MSCs in hypoxia alone has been shown to have clear translational benefits enhancing cell performance *in vitro* and *in vivo* ^41^; however, hypoxic conditioning approaches remain varied. Our findings are in line with other studies that have reported beneficial effects of hypoxia on MSC proliferation. Boregowda SV, *et. al*. previously described that atmospheric oxygen (20%) inhibits MSC proliferation and differentiation and Huang WH, *et al*. demonstrated that MSCs grown in 1% hypoxia exhibit enhanced engraftment and a 40% increase in cell survival in an ischemic environment^10,12^. Similarly, Hu X *et. al*. examined *in vivo* efficacy of MSCs that were initially grown in normoxia using a standard culture protocol^7^ and preconditioned in 0.5% oxygen and showed functional improvement to myocardial infarction in a nonhuman primate model^11^. While some studies use acutely preconditioned MSCs, others utilize MSCs grown in various degrees of hypoxia from initial isolation. Despite these differences in approach, the overarching theme remains that MSCs grown in hypoxia have improved therapeutic benefits. Hypoxia is reported to increase proangiogenic gene expression including hypoxia-inducible factor 1α (HIF-1α), angiopoietin-1 (Ang-1) and vascular endothelial growth factor (VEGF) and lowers apoptotic enzyme activity (caspase-3). However, we did not detect increases in HIF-1α protein expression in our MSCs cultured under 5% O_2_ via Western blot (not shown), potentially due to the physiologically relevant oxygen tension at which our cells were grown, which mimics the MSC bone marrow niche. This is consistent with prior studies in lung and cell culture showing minimal HIF1α induction with 5% O_2_ conditions and decaying HIF1α expression at time points longer than 4 hours^42–44^. MSCs cultured in hypoxia exhibit improved *in vivo* homing, angiogenesis and engraft better than MSCs cultured in normoxia^45^. Indeed, our own data support that growing mMSCs in 5% hypoxia from initial isolation enhanced proliferation (Figs 2 and 3), decreased senescence (Fig. 4), and allowed cells to maintain their multipotency until later passages than normoxic conditions (Fig. 5). It is well known that HIF-1α plays a central role as a low oxygen sensor after ischemic injury and that cells expressing HIF-1α are able to resist hypoxic environments and have enhanced proliferation rates. CXCR4 and CXCR7 expression in MSCs is also regulated by HIF-1α levels and stromal cell derived factor-1 (SDF-1) improves progenitor/stem cell homing and engraftment into injured sites^46,47^. Whether MSCs cultured using the hypoxia+bFGF combinatorial approach have increased regenerative potential for *in vivo* applications remains to be investigated.

Our data demonstrate that supplementation with 10 ng/mL of bFGF further enhanced hypoxia-mediated effects *in vitro*, suggesting that there is room for improvement in MSC culture methods. We demonstrated that MSCs grown in 5% hypoxia+bFGF exhibit robust regenerative potential *in vivo* (Fig. 7). Culturing MSCs in hypoxia+bFGF can improve cell expansion, allowing for the generation of a therapeutic dose of stem cells in a shorter period of time. This is a tremendous advantage in terms of stem cell manufacturing processes and may allow delivery of stem cells with low passage numbers and high quality in the clinic setting. Many reports show that bFGF treatment of MSCs promotes cell proliferation and simultaneously helps maintain tri-lineage differentiation potential. In addition, Yanada *et al*. suggested bFGF could extend MSC proliferation that can overcome the Hayflick phenomenon. MSCs cultured in bFGF alone have increased protective effects for the ischemic heart *in vivo* and enhance blood reperfusion in a pre-clinical animal model^48^. Hahn *et. al*. described cytoprotective effects with bFGF primed MSCs via enhanced gap junction formation with neighboring cardiomyocytes that was able to reduce apoptosis and increase cardiomyocyte survival after MI^49^.

Altogether, our findings clearly demonstrate that the beneficial effects of hypoxia are improved when combined with bFGF. Combinatorial use of low oxygen tension and bFGF supplementation have dramatic synergistic effects on murine MSCs, yielding rapidly proliferating MSCs that resist senescence maintain their multipotency through late passages and exhibit *in vivo* therapeutic efficacy. Compared to other mMSC isolation methods, this new synergistic approach and its dramatic effects on mMSC properties suggests there is room for improvement in MSC isolation and expansion methods to further enhance their use for pre-clinical experiments and to enhance regenerative potential *in vivo*.

## Methods

An expanded Materials and Methods section is available in the Online Data Supplement.

### Harvesting Mesenchymal Stem Cells from Mice

All animal work was approved by the Emory University Institutional Animal Care and Use Committee (IACUC) and carried out according to the guidelines. mMSCs were isolated from 6 week old male C57Bl/6 mice (Jackson Laboratories, Bar Harbor, ME). Mice were sacrificed and femurs and tibias were harvested. The marrow was flushed from the bones using 21-gauge needle and 3 mL syringe (BD Medical, Franklin Lakes, NJ, cat # 309585) using complete RPMI 1640 (Life Technologies, Carlsbad, CA, cat# 22400-105) growth media supplemented with 20% Premium Select Grade Fetal Bovine Serum (FBS; Atlanta Biologicals, Norcross, GA, cat# S11550), 2 mM glutamine (cat# 25030-081), 100 units/mL penicillin, and 100 μg/mL streptomycin (cat# SV30010), all from Life Technologies. Marrow was pipette into a homogeneous cell suspension and filtered through a 70-µm nylon mesh filter (BD Falcon, Franklin Lakes, NJ, cat# 352350) prior to plating. Additional details can be found in the Online Data Supplement.

### Plating and Culture of Mesenchymal Stem Cells

Viable cells were plated at 500,000 cells/cm^2^ in 40 mL of growth media ±10 ng/mL bFGF (Austral Biologicals, San Ramon, CA, cat# GF-030-5) per T-175 culture flask (Nunclon®, Naperville, IL, cat# 178883). After plating, MSCs were immediately transferred to incubators at 37 °C, 5% CO_2_ and either 20% oxygen (normoxia) or 5% oxygen (hypoxia). 24 hours after initial plating, the media containing any non-adherent cells was carefully removed and replaced with 30 mL of fresh growth media ±10 ng/mL bFGF. Media was changed every 3–4 days. See details on mMSCs passaging in the Online Data Supplement. When MSCs reach p2-3, basal media for cell culture changes over from RPMI to DMEM with same supplementation.

### Cell characterization and Multipotency Assessment

#### Immunophenotypic Characterization by Flow Cytometry

500,000 to 1 × 10^6^ mMSCs were collected and transferred into amber eppendorf tubes (New England Biologicals, Ipswich, MA, cat# B9000). Cells were suspended in 1% BSA with 10 µg/mL of the following antibodies: CD44, Sca-1, CD90.2, CD13, CD31, CD34 and CD45. For additional antibody information, see Table 1. Flow cytometry analysis was performed using a FACScan (Becton Dickinson, Franklin Lakes, NJ) system and >10,000 events were measured per sample. Unstained and isotype stained cells were used as controls. All data were analyzed by flowjo software (Tree Star Inc., Ashland, OR). For size and granularity experiments, at least 50,000 events per sample were measured.

#### Differentiation Assays

To verify that MSCs cultured in hypoxia and hypoxia+bFGF differentiate similar to MSCs cultured in normoxia, MSCs between passages 3–5, unless otherwise noted, were differentiated into one of three cell lineages: adipocytes, osteoblasts or chondrocytes. See supplemental methods for complete procedures. Briefly, we used the following conditions for tri-lineage differentiation:

Adipogenesis: Cells were plated at a density of 5 × 10^4^ MSCs/cm^2^ and were cultured in complete DMEM media. The following day, media was changed StemPro Adipogenesis Differentiation media (Thermo Fisher Scientific, Waltham, MA, cat# A10070–01) and differentiation media was changed every 2–3 days for 14–21 days. To verify adipogenic differentiation, adipocytes were stained with Oil Red O when vacuoles were visible (between 14–21 days) using the staining protocol described in detail in the supplemental methods section. Lipids vacuoles will stain red.

Chondrogenesis: 5.0 × 10^5^ MSCs were added to a 15 mL conical tube and spun down at 300 g for 5 minutes. 1 mL of fresh StemPro Chondrogenesis media (Thermo Fisher Scientific, Waltham, MA, cat# A10071-01) was added to the pellet. The differentiation media was changed every 2–3 days for 14–21 days. To verify chondrogenic differentiation, the pellet is fixed and stained using a primary antibody to Type II Collagen and with DAPI for nuclear stain.

Osteogenesis: MSCs were plated at a density of 6,000 cells/cm^2^ on collagen and cultured overnight. Next day, growth media was changed to 2 mL of StemPro Osteogenisis Differentiation media (Thermo Fisher Scientific, Waltham, MA, cat# A10072-01). Osteogenic media was changed every 3–4 days for 14–21 days. Osteogenic differentiation was confirmed by staining osteoblasts at 14–21 days after the initiation of differentiation using Alizarin red S. Calcium will appear red when stained with Alizarin red S.

### Proliferation Assay

MSCs (p1) were plated at a density of 20,000 cells per well in duplicates in a 24 well plate and cells per cm^2^ were quantified at 2, 7, 10, 14 and 21 days (details in Online Data Supplement).

### Colony Forming Unity (CFU) Assay

MSCs (p9) were plated at a density of 250 cells/cm^2^ and maintained in their original hypoxic (5% O_2_) +/−10 ng/mL bFGF culture conditions for 11 days. Colonies were fixed with methanol and stained with 1% crystal violet solution (Sigma Aldrich; details in Online Data Supplement). Plates were dried and colony number was counted under a dissection microscope.

### Beta Galactosidase Staining

Cells split at p1 were plated at a density of 250,000 cells per well in duplicate in a 6 well. At 50% confluence, the media was removed and cells were fixed at 37 °C for 10 minutes in 2 mL of 0.25% Glutaraldehyde. Cells were then washed with 1X PBS and 2.7 mL of 1X PBS containing 1.3 mM MgCl_2_, 5 mM K_3_[Fe(CN)_6_], 5 mM K_4_[Fe(CN)_6_], and 0.3 mL X-Gal (Invitrogen, Carlsbad, CA, cat# I46-0551) was added to each well and incubated at 37 °C for 5 hours. Representative photos were taken using a 10x objective and Olympus 1 × 71 with a DP camera. 30 continuous phase contrast images were acquired (4x) after x-gal staining and stitched together using Photoshop for quantification of senescence for a large area. For quantification, β-gal positive cells were counted using Image J 1.40 software (National Institutes of Health, Bethesda, MD) and were expressed relative to total cell number for 3 independent experiments.

### Murine Model of Hindlimb Ischemia and Encapsulated Cell Delivery

1 × 10^6^ mMSCs were encapsulated in 1% ultrapure alginate LVG (Novamatrix; details in Online Data Supplement), as described previously^25,26^. Male osteopontin knock-out (OPN−/−) mice 8–10 weeks of age were purchased (Jackson Laboratories, Maine, USA). Mice were pre-anesthetized with 3% isoflurane in a chamber and then anesthetized with 2% isoflurane through a nose cone prior to performing hindlimb ischemia surgery, as described previously^25,50^. Briefly, hair was removed from the surgical site, the area was cleaned with saline, and disinfected with Betadine. Aseptic technique was employed. A unilateral incision was made over the left medial thigh and the superficial femoral artery and vein were ligated proximal to the deep femoral artery branch point and a second ligation was performed just proximal to the branching tibial arteries. The artery and vein were excised between the two ligation points and the skin was closed with 6–0 suture. Encapsulated mMSCs (1 × 10^6^ cells/animal) or empty capsules were delivered to a separate subcutaneous pocket parallel to the ligation compartment, as previously reported^25^. Animals received Buprenex (0.01-0.1 mg/kg, SQ) post-operatively for analgesia as needed and recovered on a heated platform. The animals were housed and cared for according to the guidelines approved by the Emory University Institutional Animal Care and Use Committee.

### LASER Doppler Perfusion Imaging

LASER Doppler perfusion imaging (LDPI) was used as an indirect measure of neovascularization and was measured over time for each treatment condition, as described previously^25,50^. Briefly, mice were anesthetized by isoflurane inhalation and scanned with the LDPI system (PIM II Laser Doppler Perfusion Imager). Perfusion of the ischemic and non-ischemic limbs (Fig. 7C) were assessed. Significant changes in ischemic limb perfusion were quantified and normalized to the non-surgical limb and presented as a perfusion ratio.

### Multivariate Analysis of Cytokine and Phospho-protein Signaling

MSC cytokines and phospho-proteins were quantified via Luminex analysis (Millipore) and normalized to total protein (Pierce BCA, Thermofisher). All D-PLSR model analysis was conducted in MATLAB (Mathworks, Natick, MA) using the partial least squares algorithm by Cleiton Nunes (available on the Mathworks File Exchange). All multiplexed signaling data were *z*-scored, and then used as the independent inputs to the algorithm. An orthogonal rotation in the LV1-LV2 plane was used to choose a new LV1 that best separated the hypoxia+bFGF condition. A Monte Carlo sub-sampling using 1,000 iterations was used to characterize standard deviation on the individual signals involved in LV1 and LV2 of the D-PLSR models. For each iteration, 80% (12/15) of the samples were randomly sampled, and a new D-PLSR model was constructed. To correct for sign reversals, each sub-sampled LV1 and LV2 was multiplied by the sign of the scalar product of the new LV and the corresponding LV from the total model. The same orthogonal rotation used for the total model was applied to the LVs from each iteration, and mean and standard deviation was computed for each signal across all iterations.

### Statistical Analysis

Results are expressed as mean ± S.E.M. from at least three independent experiments. Statistical significance for quantitative results was assessed using analysis of variance (ANOVA), followed by either a Tukey or Bonferroni Multiple Comparison post-hoc test. In some cases, a Students’ *t-*test was used to assess significance. A value of *p* < 0.05 was considered statistically significant.

### Data Availability

The datasets generated during and/or analyzed during the current study are available from the corresponding author on reasonable request.

## Electronic supplementary material

Supplemental Data File
