# Supplementary material for: A Novel Technique for Accelerated Culture of Murine Mesenchymal Stem Cells that Allows for Sustained Multipotency
Source: Sci Rep. 2017 Oct 17;7:13334. doi: 10.1038/s41598-017-13477-y (PMC5645326; doi:10.1038/s41598-017-13477-y)

A Novel Technique for Accelerated Culture of Murine Mesenchymal Stem Cells that Allows for Sustained Multipotency

Courtney M. Caroti[1†], Hyunhee Ahn[2,3†], Hector F. Salazar[1], Giji Joseph[1], Sitara B. Sankar[4,6], Nick J. Willett[2,3,4], Levi B. Wood[4,5,6], W. Robert Taylor[1,3,4] and Alicia N. Lyle[1*]

[1]The Division of Cardiology, Department of Medicine, Emory University, Atlanta, GA, USA
[2]The Department of Orthopaedics, Emory University, Atlanta, GA, USA
[3]The Atlanta Veterans Affairs Medical Center Atlanta, Decatur, GA, USA
[4]The Wallace H. Coulter Department of Biomedical Engineering, Georgia Institute of Technology and Emory University, Atlanta, GA, USA
[5]George W. Woodruff School of Mechanical Engineering, Georgia Institute of Technology, Atlanta, GA, USA
[6]Parker H. Petit Institute for Bioengineering & Bioscience, Georgia Institute of Technology, Atlanta, GA, USA
*Corresponding author: alicia.lyle@emory.edu
†These authors contributed equally to this study

Supplemental Figure Legends

Supplemental Figure 1. A. Trypsin depletion method. Large and smaller senescent mMSCs that associate with them are carefully removed from the cultures using the trypsin depletion method outlined in detail in the **Methods** section. Briefly, when passaging mMSCs, the cells are quickly rinsed once with 4mL of 0.25% trypsin-EDTA to remove residual serum-containing media and to allow the depletion of the senescent cells from the cultures. Using the light microscope, watch for the large senescent cells and any associated senescent cells (**1, 1b**) to detach from the cell surface over time (seconds). Here we show two examples of how this trypsin depletion wash promotes senescent cells to release over time (**images 1-4**) prior to the healthy, non-senescent cells (**4, 4b**). Once they begin to detach, this first trypsin "wash" is removed and the senescent cells with it. **B. High magnification images of mMSCs.** Representative 10x images of mMSCs grown in four different culture conditions at p0, day 5.

Supplemental Figure 2. Sustained stemness of mMSCs in hypoxia with bFGF supplementation. A. Proliferation assay for p12 mMSCs grown in hypoxia or hypoxia+bFGF show similar cell proliferation rates to p1 mMSCs. n=3-4, * p<0.001 vs. hypoxia. **B.** CFU-F assay

to show the colony forming capacity of p9 mMSC with and without bFGF supplementation. n=4, * $p<0.0001$ vs. hypoxia; Data presented as mean ± S.E.M.

Supplemental Figure 3. Cell population size and granularity profiles. Senescent MSCs, if allowed to persist in culture, limit the number of passages that the MSCs will grow. Therefore, we use a trypsin depletion method to deplete these senescent cells from the culture populations at the time of cell passaging (**Supplemental Figure 1**; see **Methods** for details). **A.** FACS scatter plots show the MSC population distribution at p3-4 after the careful removal of as many large senescent cells, and the underlying small senescent cells (**Sup. Fig. 1**), from the culture population as possible. Doing this substantially minimizes the size (FSC) and granularity (SSC) variability in the following mMSC cell populations: normoxia+bFGF, hypoxia, and hypoxia+bFGF. **B.** The percent of each mMSC culture population that exhibited increased granularity (Q1+Q2), increased size (Q2+Q3), or both (Q2 only) in the murine MSC cultures was quantified using the gates indicated in **Sup Fig 2A**. The depletion of these senescent cells from the culture result in MSC cultures where the majority of the MSC cells exhibit a consistent size and ~86.8% fall within the expected gated population (Q4; **sup. Fig 2A**).

Supplemental Figure 4. mMSC appearance and viability after encapsulation. A. Representative phase contrast image to show the appearance of encapsulated mMSCs at the time of implantation. **B.** The viability of mMSCs was determined prior to subcutaneous implantation using a fluorescent LIVE/DEAD assay for viability; green = viable, red = non-viable. The image shown is a maximum intensity project of capsules showing that at least 90% of cells were viable. Capsules are 250 µm in diameter.

Supplemental Figure 5. Vertical axes for D-PLSR signaling analyses. The vertical axis separates hypoxia from normoxia conditions in both the cytokine and phospho-protein models. **A.** LV2 consists of a linear combination of cytokines that correlate with normoxia (negative) or hypoxia (positive) conditions (mean±SD, 1000 Monte Carlo iterations, 20% sample removal). **B.** LV2 consists of a linear combination of phospho-proteins that correlate with normoxia (negative) or hypoxia (positive) conditions (mean±SD, 1000 Monte Carlo iterations, 20% sample removal).

Materials and Methods

Serum Selection

Premium Select Fetal Bovine Serum (FBS-PS), triple 0.1 µm filtered, was purchased from Atlanta Biologicals (Atlanta Biologicals, Norcross, GA, cat# S11550). The biological composition of the FBS lot can make a tremendous difference when growing mMSCs. We initially selected an appropriate FBS-PS lot by using a standard proliferation assay to determine what lots gave the best growth curves for our mMSC cultures grown under normoxic conditions. This was done by plating 20,000 cells per well of a 24 well plate (in duplicate) and harvesting and counting the cells at days 2, 7, 10, 14, and 21. Future FBS-PS lots were then screened and selected in a similar fashion; however, FBS lots biologically similar to the lot our lab initially identified were requested and then used for all additional experiments. Other qualitative measures used to determine lot selection included: colony compactness and cell morphology and lack of increased senescence. When choosing a new lot, we always request two new lots biologically similar to the lot our lab is currently using and use the proliferation assay to compare how our cells grow in the new FBS-PS lots compared to the current lot. The most current lot screened and found to be appropriate for mMSC culture is FBS-PS lot# L14148.

Harvesting Mesenchymal Stem Cells from Mice

All animal work was approved by the Emory University Institutional Animal Care and Use Committee (IACUC) and carried out according to the guidelines. mMSCs were isolated from 6 week old male C57Bl/6 mice purchased from Jackson Laboratories (Bar Harbor, ME). C57Bl/6 mice were sacrificed and the femurs and tibias were harvested. After isolation and cleaning, the marrow was flushed from the bones using 21-gauge needle and 3 mL syringe (BD Medical, Franklin Lakes, NJ, cat # 309585) into complete RPMI 1640 (Life Technologies, Carlsbad, CA, cat# 22400-105) growth media supplemented with 20% Premium Select Grade Fetal Bovine Serum (FBS; Atlanta Biologicals, Norcross, GA, cat# S11550), 2 mM glutamine (cat# 25030-081), 100 units/mL penicillin, and 100 µg/mL streptomycin (cat# SV30010), all obtained from Life Technologies. Cells were gently pipetted into a single cell suspension and filtered through a 70-µm nylon mesh filter (BD Falcon, Franklin Lakes, NJ, cat# 352350) into a fresh, sterile tube.

Plating and Culture of Mesenchymal Stem Cells

Viable cells were plated at 500,000 cells/cm^2 in 40 mL of growth media per T-175 culture flasks (Nunclon®, Naperville, IL, cat# 178883). The growth media of bFGF groups was supplemented

with 10 ng/mL bFGF (Austral Biologicals, San Ramon, CA, cat# GF-030-5). After plating, MSCs were immediately transferred to incubators at 37˚C, 5% CO_2 and either 20% oxygen (normoxia) or 5% oxygen (hypoxia). 24 hours after initial plating of the bone marrow derived cells, the media containing any non-adherent cells was carefully removed and replaced with 30 mL of fresh growth media +/- 10 ng/mL bFGF. Each media was changed every 3-4 days. We observed that cells grow better changing the media at this frequency compared to changing media every 2-3 days. See details on mMSCs passaging below. When MSCs reach p2-3, basal media for cell culture changes over from RPMI to DMEM with same supplementation.

Passage of mMSCs

p0 → p1

Cells were grown to the appropriate level of confluence prior to passage. To passage mMSCs from p0 → p1, culture media was removed and the cells were quickly rinsed once with 4mL of 0.25% trypsin-EDTA (Life Technologies, Gaithersburg, MD, cat# 25200114) by rolling the trypsin over the cell surface to help remove any residual serum-containing growth media. The flask of cells was then carried over to a light microscope and the trypsin wash was allowed to cover the cell surface for a slightly longer amount of time to allow the senescent cells from the cultures. Using the light microscope, watch for the large senescent cells and any associated senescent cells (see Figure 4A for examples) to detach from the cell surface. As we show in Supplemental Figure 1A, this initial trypsin depletion wash is left on the cell surface to allow for these senescent cells and associated cells to release. Once they begin to detach, this first trypsin “wash” is removed and the senescent cells with it. 4 mL of fresh trypsin 0.25% trypsin-EDTA is then added to the flask to detach all remaining cells, which should take ~2-5 minutes in the 37°C incubator. Once cells are all nearly detached, rap the flask firmly 2-3 times max to detach any remaining cells from the flask surface. Add 16 mL of complete RPMI growth media to neutralize the trypsin and pipette the cell suspension up and down to break apart clumps and dissociate into individual cells. Transfer the entire 20 mL suspension into a sterile 50 mL conical tube and centrifuge cells at 300xg for 10 minutes at room temperature. After centrifuging, remove media and resuspend the cell pellet in 4 mL of complete RPMI. Viable cells should then be counted using trypan exclusion (as described earlier in the protocol). Typically, at this point we don’t split our mMSCs wider than 1:2; however, cells can *plated at a density of* 500,000 cells/cm^2 in a T-175 flask. We find this heavy density helps to minimize mMSC senescence.

p1 → p2

After the first split, the media +/- 10 ng/mL bFGF should be changed the following day to remove cells that have not adhered to the bottom of the flask. Adherent p1 cells should be fed with complete RPMI growth media +/- 10 ng/mL bFGF every 3-4 days. When cells are just sub-confluent (~90-95%), cells should be split as outlined above, again using the trypsin depletion method to remove senescent cells from the culture. Cells should be split 1:2 once more.

p2 → p3

Adherent p2 cells should be fed with complete RPMI growth media +/- 10 ng/mL bFGF every 3-4 days. When cells are just sub-confluent (~90-95%), cells should be split as outlined above, again using the trypsin depletion method to remove senescent cells from the culture. Cells should be split and plated at p3 at a minimal density 3,000 cells/cm^2 in a T-175 flask. 24 hours after plating cells at p3, the media can be changed over from complete RPMI growth media to complete DMEM growth media and cultured in DMEM (Sigma Aldrich, St. Louis, MO, cat# D5671) basal media supplemented with 20% FBS-PS, 2 mM glutamine (cat# 25030-081), 100 units/mL penicillin and 100 µg/mL streptomycin (cat# SV30010), and HEPES (HyClone, Ogden, UT, cat# SH30237.01). MSCs should be fed with complete DMEM growth media +/- 10 ng/mL bFGF every 3-4 days. Continue to feed and culture cells for subsequent passages by using the trypsin depletion method, if necessary, and plate cells at a density of 3,000 cells/cm^2 at each passage.

p3+

mMSCs from p0 to p2 appear as rounded, refractory cells that rapidly proliferate. We wanted to take the time to discuss here that there will be a transition period for the cells between p2 and p4. During this time, one will notice that the mMSC morphology begins to change from the rounded appearance to a more spindle-shaped morphology. During this transition phase, the cells proliferation rate can also slow somewhat, so do not be alarmed if this occurs. Continue to feed cells as usual (~3-4 days) until they complete this phenotypic transition.

After p3-4 MSCs have made the phenotype transition and reach sub-confluence, they are then ready for the following:

- Immunophenotyping by Flow Cytometry Analysis
- Differentiation Assays for Multipotency Assessment
- Any additional uses

Cell characterization and Multipotency Assessment

Immunophenotypic Characterization by Flow Cytometry

500,000 to $1x10^6$ mMSCs were collected and transferred into amber eppendorf tube (New England Biologicals, Ipswich, MA, cat# B9000). Cells were then resuspended in 1% BSA with a concentration of 10 µg/mL of the following antibodies: CD44, Sca-1, CD90.2, CD13, CD31, CD34 and CD45 respectively. For additional antibody information, see **Table 1**. Flow cytometry analysis was performed using a FACScan (Becton Dickinson, Franklin Lakes, NJ) system and at least 10,000 events per sample were measured. Unstained and isotype stained cells were used as controls. All data were analyzed by FlowJo software (Tree Star Inc., Ashland, OR). For size and granularity experiments, at least 50,000 events per sample were measured.

Differentiation Assay

Cell Preparation for Differentiation Assays

To verify that MSCs cultured in hypoxia and hypoxia+bFGF differentiate the same as MSCs cultured in normoxia, MSCs between passages 3-5, unless otherwise noted, were differentiated into one of three cell lineages: adipocytes, osteoblasts or chondrocytes. First, cell culture media is removed and cells were washed with 4 mL of TrypLE (Thermo Fisher Scientific, Waltham, MA, cat# 12605028). A fresh 4 mL of TrypLE was added and flasks were placed in the 37˚C hypoxia incubator for 2-3 minutes for cells to round up and/or detatch. To completely dislodge cells from the flask surface, the flasks were gently rapped. 12 mL of complete RPMI was added to the flask to neutralize TrypLE and the 16 mL cell suspension was transferred to a conical tube and centrifuged at 300 xg for 10 minutes. Cells were resuspended in complete RPMI and counted using the Trypan exclusion procedure described above. MSCs were then used at the designated densities outlined below for adopogensis, chrondrogenisis or osteogensis differentiation.

Adipogenesis

Cell Culture: Cells were plated in 6 well plates (BD Falcon, Franklin Lakes, NJ, cat# 877229) at a density of 9.4x10^4 cells per well in a total volume of 3 mL per well and were cultured in complete DMEM in 20% oxygen, 5% CO_2 at 37˚C. The following day, growth media was removed and changed to 3mL of complete StemPro Adipogenesis Differentiation media (kit from Thermo Fisher Scientific, Waltham, MA, cat# A10070-01) and negative control wells were given 3 mL of fresh complete DMEM. Media for both groups was changed once every 2-3 days for 14-21days.

Cell Staining Protocol for Oil Red O: Adipocyte staining with Oil Red O was performed when vacuoles started to form (between 14-21 days) using the Lonza staining protocol.

Fixing Cells: The cells were gently washed with 2 mL of sterile 1x DPBS without disturbing the cell monolayer before being fixed for 30 minutes at room temperature in 2 mL of 10% Formalin per well.

Preparing Oil Red O Stain: The Oil Red O stock solution is made by combining 300 mg of Oil Red O powder with 100 mL of 99% Isopropanol. This stock solution is stable for 1 year from date of preparation. In the fume hood, the Oil Red O working solution is prepared by mixing 3 parts (30 mL) of Oil Red O stock solution with 2 parts (20 mL) dH2O and allowed to sit at RT for 10 minutes. Once the working solution is prepared, it is stable for no longer than 2 hours and should be filtered through Whatman No. 1 filter paper prior to use.

Staining Adiopogenic Cultures: After the cells have been fixed, slightly tilt the plate and remove the formalin. Gently add 2 mL of sterile dH2O to each well to rinse the monolayer. Aspirate the water and add 2 mL of 60% Isopropanol to cover the bottom of each well and let sit for 2-5 minutes. Aspirate Isopropanol and add 2 mL of the working Oil Red O solution to each well so that the cell monolayer is completely covered and allow to stand for 5-7 minutes at room temperature. Rinse the cells with tap water using a Pasteur pipette until the water runs clear. Then, pipet 2 mL of the hematoxylin counterstain (cat# 72604) to each well so that cells are completely covered and allow to stand for 1 minute at room temperature. Aspirate off hematoxylin and rinse with tap water until the water runs clear. Keep the plates wet with water until they are ready to be imaged using a phase contrast microscope. The lipids will appear red and nuclei are blue.

Chondrogenesis

Cell Culture: After counting, 5.0×10^5 cells were placed in a 15 mL vial and spun down at 300xg for 5 minutes. The media was then aspirated and cells were washed by resuspending pellet in 1 mL of complete StemPro Chondrogenesis Differentiation media (Thermo Fisher Scientific, Waltham, MA, cat# A10071-01). Cells were then spun down at 300g for 5 minutes and media is aspirated. 1 mL of fresh chondrogenic media was added to the pellet and the pellet was incubated over night at 37°C, 5% CO_2, and 20% oxygen in 15 mL tubes stored upright with caps loose to allow air exchange. The following day, the pellet was spun at 300g for 5 minutes and the old media was aspirated and changed to 1 mL of fresh chrondrogenic media. It is important to tilt tube to side and avoid aspirating the pellet. After the media was changed, the tube was gently

flicked until the pellet was dislodged/released from the bottom of the tube and floating. One must be cautious to release the pellet without breaking up the cell pellet.

Media change: Media should be changed 24 hours after initiation of the differentiation protocol and then every 2-3 days to keep media from becoming too acidic. Each time the media is changed, cells are spun at 300g for 5 minutes to allow pellet to move to bottom of the tube. Old media is then aspirated with 200 µl tip on tubing and by carefully titling the tube to the side to avoid disrupting or aspirating the pellet. 1 mL of fresh chondrogenesis media is added to the tube and then the tube is flicked at the bottom to gently loosen pellet after media change. Cells will begin secreting collagen, which will make the media in the tube appear cloudy, and should be stained after 14-21 days of differentiation.

Cell Staining Protocol for Chondrocytes: Between 14 and 21 days post-initiation of the chondrogenesis differentiation protocol, the pellet is removed from media using a Pasteur pipette, wrapped in bio-wrap (cat# 01090), and placed into a tissue cassette. The pellet is then fixed in 4% formaldehyde for 30 minutes and then placed in 70% ethanol overnight at 4˚C. The next day, pellets were dehydrated in successive ethanol washes of 70%, 70%, 80%, 80%, 95% and 95%, for 20 minutes each. Next, pellets were transferred to xylene, two changes, 20 minutes each and then transferred to three changes of 58°C paraffin, 20 minutes each. Pellets were then removed from histology cassette and paper and embed in a paraffin block as per standard embedding procedures. The blocks were then cut in 10 µm sections of each pellet and transfer to a 40°C water bath. Sections of each pellet were then placed onto positively charged glass microscope slides and then stored the slides at room temperature for future staining. Slides were deparaffinized in xylene (3 x 3 min), 100% ethanol (2 x 3 min), 95% ethanol (2 x 3 min), 70% ethanol (1 x 3 min) then deionized water for 5 minutes. Next, endogenous peroxidase activity is quenched by incubating the pellet sections with Peroxidase Block (Dako, Atlanta, GA, cat# X0909) or, followed by 2X5 minute washes in deionized water and wash buffer (Tris buffered saline, 0.05% Tween) Pellet sections were then treated with proteinase K for five minutes, followed by 2x5 minute washes in deionized water. Sections were then incubated with serum-free protein block (DAKO corporation, Carpinteria, CA, cat# X0909) for thirty minutes, then washed with 1XPBS 3x5 minutes. Incubate sections with an appropriately characterized primary antibody to Type II Collagen for 30 minutes (controls incubated in 3% BSA for 30 minutes), then wash with buffer. Incubate sections with the appropriate peroxidase labeled secondary reagents and substrate-chromogen (peroxide-DAB) as per kit instructions. Counterstain with hematoxylin and appropriate bluing agent. Mount section in synthetic resin. Pellet sections were then viewed

using a Zeiss Axiocam microscope taking 4X and 10X images with Type II Collagen stained red and nuclei stained blue.

Osteogenesis

Cell Culture: Before plating cells, each well of a 6 well plate was collagen coated with 3 mL of 0.1% Type1 Collagen (Sigma cat#C8919) in 0.2 N of acetic acid for 30 minutes. The remaining collagen solution was then aspirated and wells were rinsed 2 x 3 mL with complete DMEM to remove residual acetic acid. Cells were plated at a density of 6,000 cells/cm^2 in a total volume of 3 mL of complete DMEM and placed in 5% CO_2 37˚C, 20 % oxygen incubator for differentiation process. 24 hours after plating, the growth media for half of the wells were changed to 2 mL of StemPro Osteogenisis Differentiation media (Thermo Fisher Scientific, Waltham, MA, cat# A10072-01), while the other half were given complete DMEM as a control. Media is changed every 3-4 days and cells were allowed to grow for 14-21 days.

Cell Staining using Alizarin Red S: Osteoblast staining was done 14-21 days after the differentiation process was initiated using Alizarin red S. Osteoblast cells will begin secreting calcium, which may make the media appear cloudy.

Preparing Alizarin red S: 2% Alizarin Red S solution is made by adding 2 g of Alizarin Red S (Sigma–Aldrich, St. Louis, MO, cat# A5533) to 100 ml of dH_2O. After the solution was mixed, the pH was adjusted to 4.2 with ammonium hydroxide. After 14-21 days of differentiation, the media was removed from the cell monolayer and the cells were rinsed once with 1x DPBS. Cells were then fixed in 4% formalin for 30 minutes and, after fixation, rinsed twice with dH_2O. All wells were then stained with 2% Alizarin Red S pH 4.2 for 3 minutes. After staining, the cells were rinsed 3 times with dH_2O and then kept wet with dH_2O. Cells were then imaged using a phase contrast microscope.

Proliferation Assay

Cells split at p1 were plated at a density of 20,000 cells per well in duplicates in a 24-well plate for the following time points: 2, 7, 10, 14 and 21 days. At each time point, cells per cm^2 were counted. Cells were maintained at 5% CO_2 37˚C in normoxic or hypoxic conditions and media changed every 3-4 days with complete RPMI and +/-10 ng/mL bFGF. For cell counting, the media was removed from each well and cells in each well were quickly washed with 200 µL of 0.25% trypsin to remove residual FBS. 200 µL of fresh trypsin was then added to each well and cells were incubated at 37˚C for 3-5 minutes or until cells detach. Once most of the cells have detached, 200 µL of complete RPMI is added to neutralize the trypsin and a cell lifter is used to gently lift the

remainder of the cells. 50 µL of cell suspension is added to 50 µL of Trypan Blue and cells were quantified using the same Trypan Blue exclusion method described above. The duplicate well cell counts were averaged to get the final cell number at each time point and the experiment was performed 3-4 independent times.

Beta Galactosidase Staining

Cells split at p1 were plated at a density of 250,000 cells per well in duplicate in a 6 well plate and were cultured until cells reached 50% confluence, at which time the media was removed, rinsed with 1X PBS, and 2 mL of 0.25% Glutaraldehyde was used to fix cells at 37˚C for 10 minutes. Cells were then washed 4 x 5 minutes with 1X PBS. 2.7 mL of 1X PBS containing 1.3mM $MgCl_2$, 5mM $K_3[Fe(CN)_6]$, 5mM $K_4[Fe(CN)_6]$ was mixed with 0.3 mL X-Gal (Invitrogen, Carlsbad, CA, cat# I46-0551) and added to each well. Cells were incubated at 37˚C for 5 hours (optimal time for mMSCs) and representative photos were taken using a 10x objective and Olympus 1X71 with a DP camera. 30 continuous phase contrast images were acquired (4x) after x-gal staining and stitched together using Photoshop for quantification of senescence for a large area. For quantification, β-gal positive cells were counted using Image J 1.40 software (National Institutes of Health, Bethesda, MD) and were expressed relative to total cell number for 3 independent experiments.

Colony Forming Unity (CFU) Assay

MSCs (p9) were plated at a density of 250 cells/cm^2. Cells were maintained in their original hypoxic (5% O_2) culture conditions at 37˚C. Cell media was changed every 3-4 days to fresh growth media +/-10ng/mL bFGF. At day 11, cells were visualized using crystal violet staining. Briefly, cells were fixed with methanol for 3 mins and washed twice with PBS. 1mL of 1% crystal violet solution (cat# v5265, Sigma Aldrich) was then added to each well and incubated for 2 min. Crystal violet staining solution was removed and the plate was rinsed with PBS until excess dye was removed. Plates were dried and colony numbers were counted under a dissection microscope. Colonies with a minimum of 50 cells were enumerated.

Cell Encapsulation and Viability Staining

1×10^6 mMSCs per animal were resuspended in 100 µL of 1% ultrapure low viscosity sodium alginate LVG (Novamatrix) and were encapsulated, as described previously[17,18], by using an electrostatic encapsulator (Nisco) with a 0.17 mm nozzle at a flow rate of 10 mL/h and 7 kV voltage, and were gelled in a solution of 50 mmol/L $BaCl_2$. Encapsulated mMSC were then

washed in 0.9% saline prior to implantation to remove residual $BaCl_2$. Encapsulated cell viability was confirmed post-encapsulation using a LIVE/DEAD Viability/Cytotoxicity Kit (Invitrogen, Carlsbad, CA, cat# L3224). Phase contrast images of the encapsulated cells are presented in Supplemental Figure 4 and the relative number of calcein-AM stained live cells (green) to ethidium homodimer stained dead cells (red) was assessed by serial z-series images obtained by confocal microscopy. Images were presented as a maximum intensity projection.

Murine Model of Hindlimb Ischemia and Encapsulated Cell Delivery

Male osteopontin knock-out (OPN-/-) mice 8-10 weeks of age were purchased (Jackson Laboratories, Maine, USA). Mice were pre-anesthetized with 3% isoflurane in a chamber and then anesthetized with 2% isoflurane through a nose cone prior to performing hindlimb ischemia surgery, as described previously[25,49]. Briefly, all hair was removed from the surgical site, the area was cleaned with sterile saline, and disinfected with Betadine. Aseptic technique was employed. A unilateral incision was made over the left medial thigh and the superficial femoral artery and vein were ligated with 6-0 suture proximal to the deep femoral artery branch point and a second ligation was performed just proximal to the branching of the tibial arteries. The length of the artery and vein were excised between the two ligation points and the skin was closed with 6-0 suture. mMSCs were encapsulated as described previously[25,26] and 1×10^6 encapsulated mMSCs or empty capsules without cells were delivered subcutaneously to a separate incision site running parallel to the ligation compartment, as previously reported[25]. Viable encapsulated mMSCs were delivered into this separate subcutaneous pocket at the time of hindlimb ischemia surgery and the skin of the pocket containing encapsulated mMSCs was closed using 6-0 suture. The animals received Buprenex (0.01-0.1mg/kg, SQ) post-operatively for analgesia as needed and were allowed to recover on a heated platform. The animals were housed and cared for according to the guidelines approved by the Emory University Institutional Animal Care and Use Committee.

LASER Doppler Perfusion Imaging

LASER Doppler perfusion imaging (LDPI) was used as an indirect measure of neovascularization and was measured over time (days) post-surgery for each treatment condition, as described previously[25,49]. Briefly, mice were anesthetized using inhalation of isoflurane and scanned with the LDPI system (PIM II Laser Doppler Perfusion Imager). Perfusion of the ischemic and non-ischemic limbs (Fig. 7C) just below the proximal ligation point were assessed. Significant changes in ischemic limb perfusion were quantified and normalized to the same region of the non-surgical limb and presented as a perfusion ratio.

Multivariate Analysis of Cytokine and Phospho-protein Signaling

MSC Cytokines and phospho-proteins were quantified via Luminex analysis (Millipore) and normalized to total protein in each well (Pierce BCA, Thermofisher). All D-PLSR model analysis was conducted in MATLAB (Mathworks, Natick, MA) using the partial least squares algorithm by Cleiton Nunes (available on the Mathworks File Exchange). All multiplexed signaling data were z-scored, and then used as the independent inputs to the algorithm. An orthogonal rotation in the LV1-LV2 plane was used to choose a new LV1 that best separated the hypoxia+bFGF condition. A Monte Carlo sub-sampling using 1,000 iterations was used to characterize standard deviation on the individual signals involved in LV1 and LV2 of the D-PLSR models. For each iteration, 80% (12/15) of the samples were randomly sampled, and a new D-PLSR model was constructed. To correct for sign reversals, each sub-sampled LV1 and LV2 was multiplied by the sign of the scalar product of the new LV and the corresponding LV from the total model. The same orthogonal rotation used for the total model was applied to the LVs from each iteration, and mean and standard deviation was computed for each signal across all iterations.

Statistical Analysis

Results are expressed as mean ± S.E.M. from at least three independent experiments. Statistical significance for quantitative results was assessed using analysis of variance (ANOVA), followed by either a Tukey or Bonferroni Multiple Comparison post-hoc test. In some cases, a Students' *t*-test was used to assess significance. A value of $p<0.05$ was considered statistically significant.

Data Availability

The datasets generated during and/or analyzed during the current study are available from the corresponding author on reasonable request.

Supplemental Figure 1.

A.

Trypsin Depletion Method

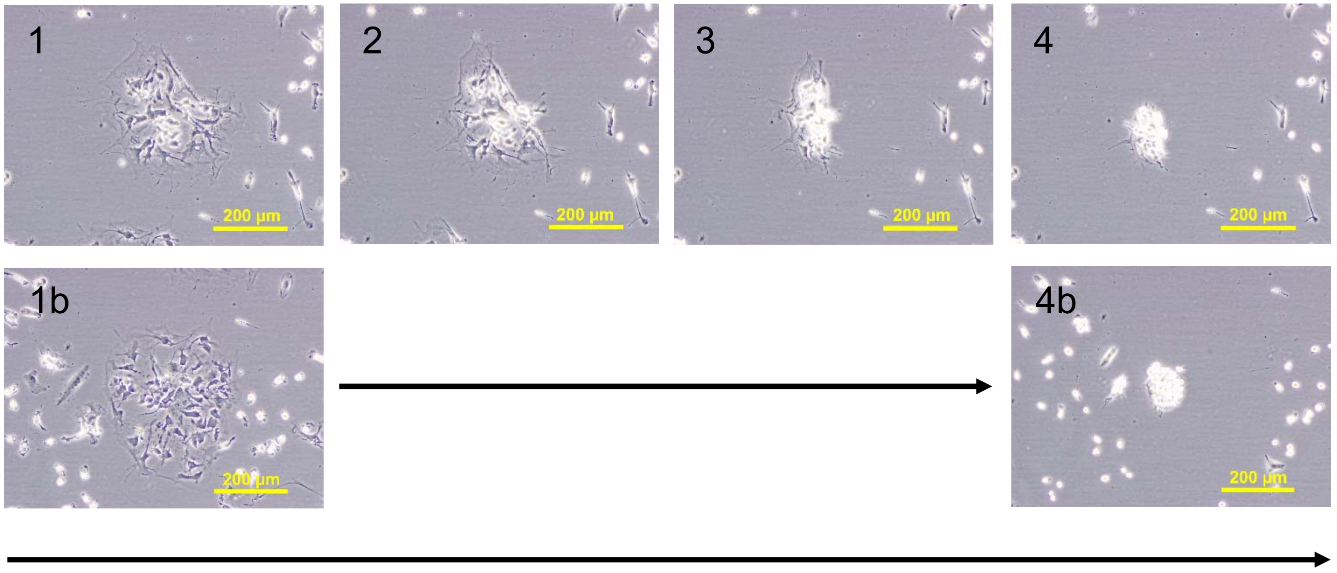

Time

B.

Normoxia | Normoxia + bFGF | Hypoxia | Hypoxia + bFGF

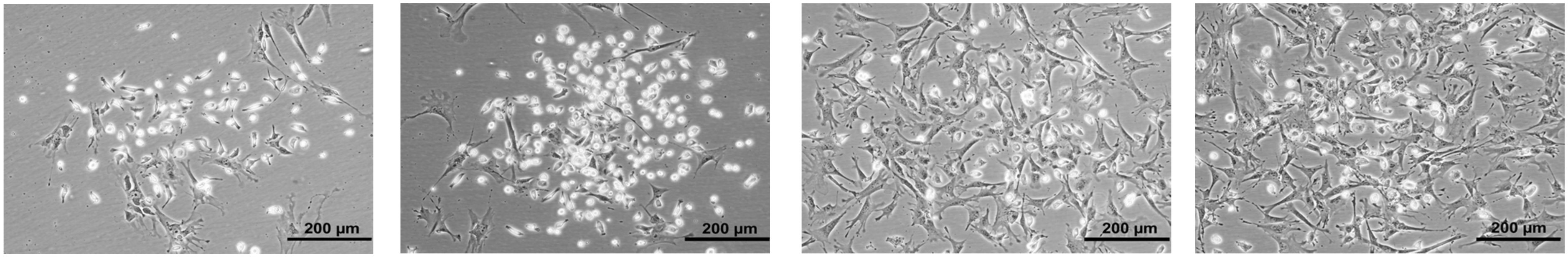

Supplemental Figure 2.

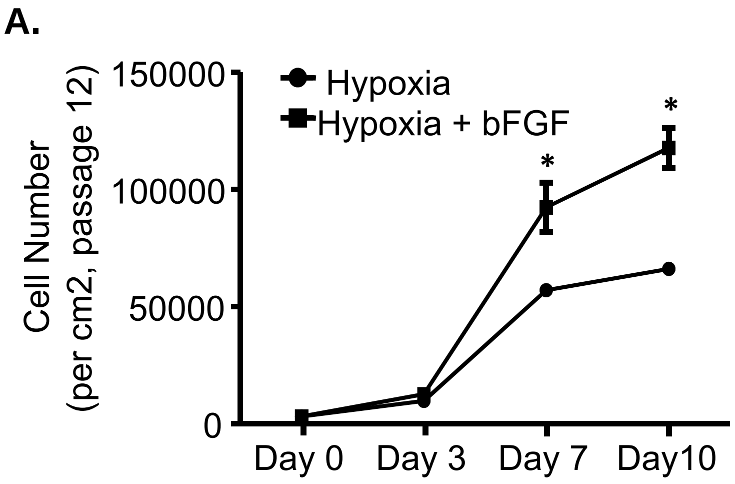

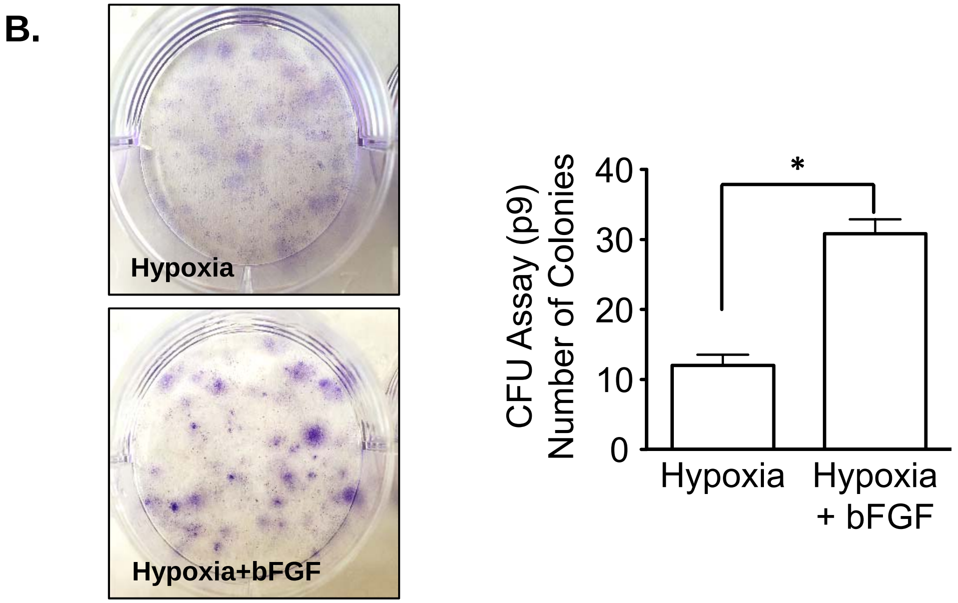

Supplemental Figure 3.

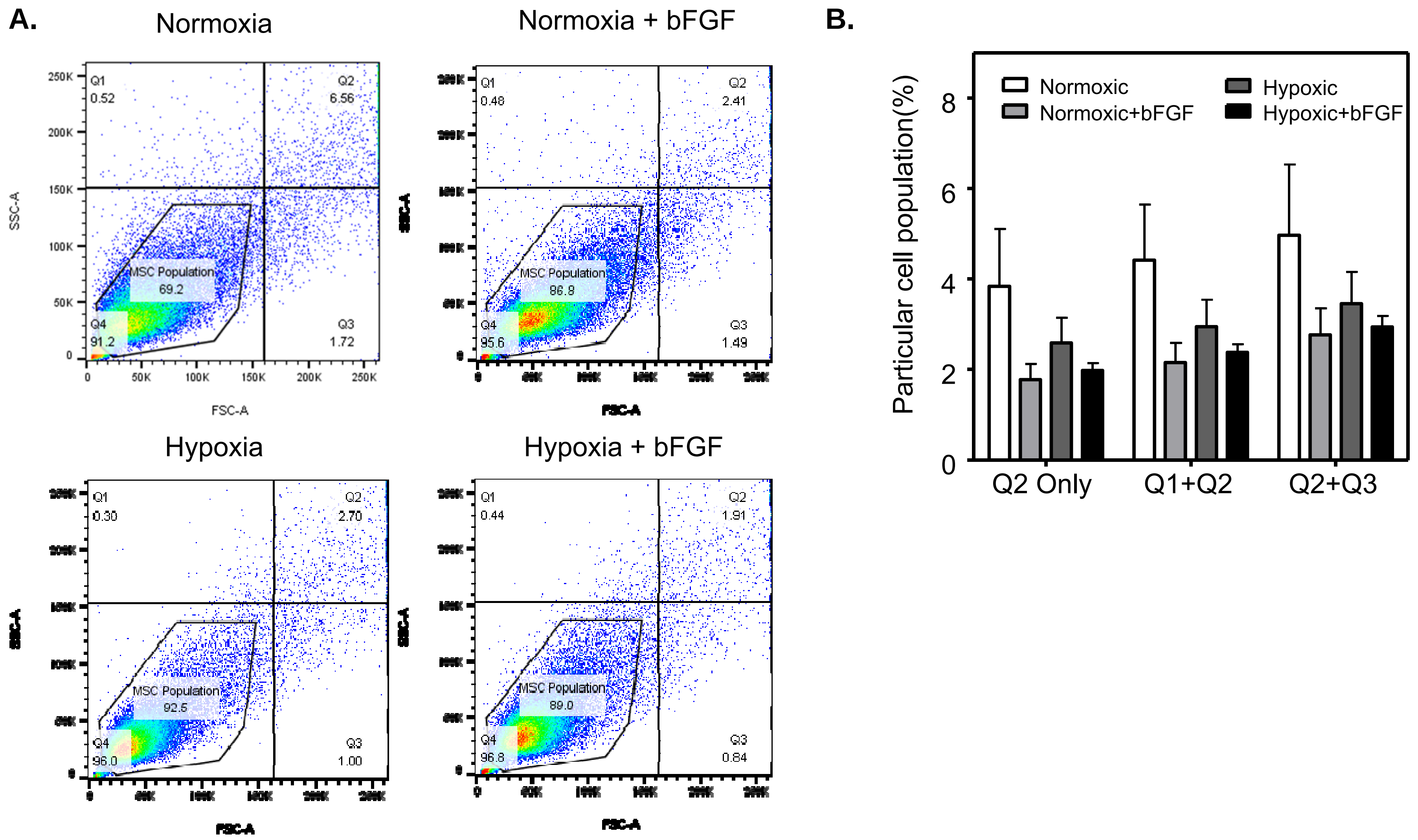

Supplemental Figure 4.

A. **B.**

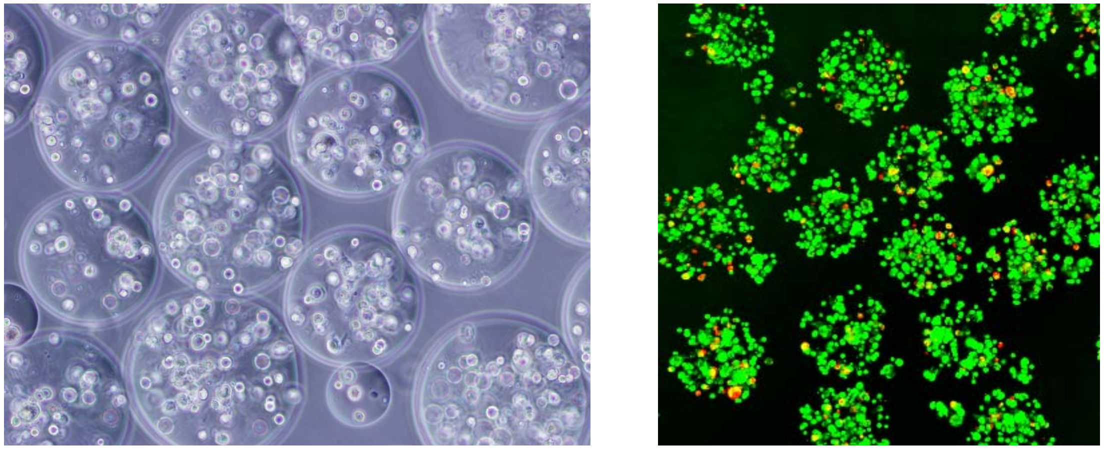

Supplemental Figure 5.

A.

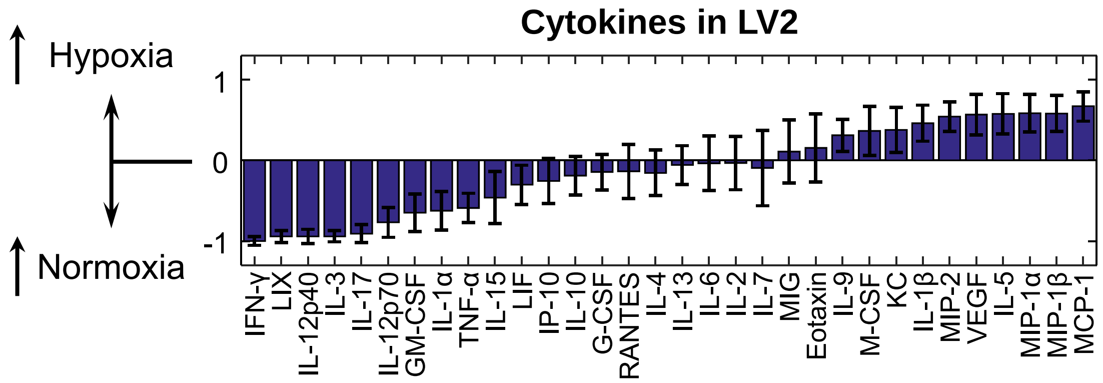

B.

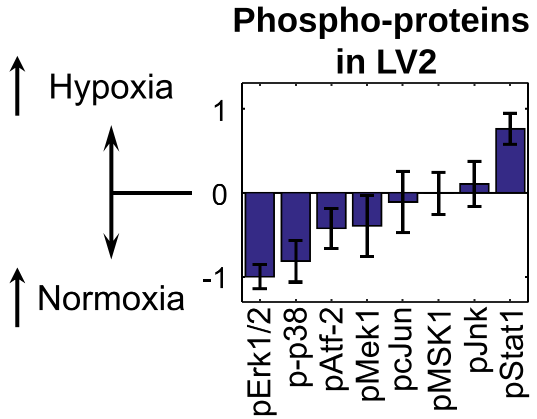

Supplement: Supplementary file 1 — Supplemental Data File [file 41598_2017_13477_MOESM1_ESM.pdf]
